# What drives compliance with COVID‐19 measures over time? Explaining changing impacts with Goal Framing Theory

**DOI:** 10.1111/rego.12440

**Published:** 2021-10-07

**Authors:** Frédérique Six, Steven de Vadder, Monika Glavina, Koen Verhoest, Koen Pepermans

**Affiliations:** ^1^ Research Group Politics and Public Governance University of Antwerp GOVTRUST Centre of Excellence Antwerp Belgium; ^2^ Department of Political Science and Public Administration Vrije Universiteit Amsterdam Amsterdam The Netherlands; ^3^ Faculty of Social Sciences, Research Group Politics and Public Governance University of Antwerp GOVTRUST Centre of Excellence Antwerp Belgium; ^4^ Faculty of Law, Research Group Government and Law University of Antwerp GOVTRUST Centre of Excellence Antwerp Belgium; ^5^ University of Antwerp Antwerp Belgium

**Keywords:** compliance, COVID‐19, fear, Goal Framing Theory, social contagion, trust

## Abstract

The COVID‐19 pandemic provides a unique opportunity to study which factors drive compliance and how the evolving context in society –virus fluctuations and changing government measures – changes the impact of these factors. Extant literature lists many factors that drive compliance – notably enforcement, trust, legitimacy. Most of these studies, however, do not look across time: whether a changing context for citizens changes the impact of factors driving compliance. In this study, we use Lindenberg's Goal Framing Theory to explain the dynamics of these drivers of compliance during the COVID‐19 pandemic. We formulate hypotheses for pro‐socialness, trust in government, observed respect for rules, rule effectiveness, rule appropriateness, fear of COVID‐19 (severity and proximity), opportunities for pleasure and happiness, as well as worsened income position. We test our hypotheses with data collected at three different moments during the beginning of the COVID‐19 crisis in Flanders, Belgium. Findings show that over time the constellations of factors that drive compliance change and, later in the pandemic, more distinct groups of citizens with different motivations to comply are identified. The overall conclusion is that the voluntary basis for compliance becomes more fragile over time, with a more differentiated pattern of drivers of compliance emerging. Public policy and communication need to adapt to these changes over time and address different groups of citizens.

## 1 Introduction

During the COVID‐19 crisis, the behavior of citizens is crucial in stopping the spread of the coronavirus: only if citizens are willing to actually change their behavior and comply with the new rules to inhibit the viral contagion, will it be possible to sufficiently contain the negative side effects on the health care system, the economy and the social fabric of society. At the start of the crisis in Europe, in March 2020, many governments imposed severe measures on their citizens to stop the spread of the virus in society. During this first peak of COVID‐19 infections, streets were empty and the level of compliance was high. Since then, compliance seems to be much lower at times when (more local) outbreaks required the reintroduction of stricter measures. It is, therefore, of high practical relevance for policy makers to better understand the dynamics of compliance over time and the changing impact of factors that influence rule compliance.

The rules that governments introduce in the COVID‐19 crisis cannot be effectively enforced by monitoring and sanctioning alone. Such coercive compliance will only lead to high enough compliance if the sanctions are severe enough and the risk of getting caught high enough (Becker 1968; Tenbrunsel & Messick 1999; Trinkner & Tyler 2016). And in most (democratic) countries this is not realistic. What is, therefore, needed for the successful reduction of the virus is voluntary, obligation‐based compliance. In other words, people need to feel the obligation to voluntarily comply with the rules even if it takes an effort (Tyler 2006).

Extant literature shows convincing evidence that, for example, trust in the regulator – in our study the government – is an important factor that stimulates this obligation‐based compliance during normal, non‐crisis conditions (e.g. Murphy 2004; Gunningham & Sinclair 2009; Six & Verhoest 2017); and in times of crisis (e.g. Tang & Wong 2003; Rubin *et al*. 2009; Blair *et al*. 2017; Vinck *et al*. 2019). Several studies have shown that also in the COVID‐19 crisis trust in government is an important driver of rule compliance (Bargain & Ulugbek 2020; Brouard *et al*. 2020; Devine *et al*. 2020; Jørgensen *et al*. 2020). Other factors are also at play like fear or pro‐social attitudes. Most of these studies, however, do not look across time to explore whether the changing context for citizens changes the impact of factors influencing rule compliance.

Which theories on compliance look at the dynamics over time? This temporal dynamic is absent in Tyler's (2006) theory of why people obey the law, with its focus on procedural justice, and in van Rooij's theory identifying motivational and situational factors as drivers of compliance (see van Rooij *et al*. 2020). Braithwaite's Responsive Regulation Theory looks at the dynamics within the relationship between regulator and regulatee, but not at the influence of contextual factors as they change over time (Ayres & Braithwaite 1992; Braithwaite *et al*. 2007). Lindenberg's Goal Framing Theory (GFT) takes a dynamic view on rule compliance and it is most explicit in how it incorporates the impact of changing context factors on compliance and why these factors drive compliance (Lindenberg 2006, 2017; Lindenberg & Steg 2007; Etienne 2013; Lindenberg *et al*. 2021). This is precisely what is needed if we want to explain the phenomena observed during the COVID‐19 pandemic: the suddenly erupting crisis followed by the varying prevalence of infected citizens and evolving knowledge about the virus combined with a wide range of government measures introduced to tackle changes in the spread of the virus. The research question in this article is, what drives rule compliance with COVID‐19 measures over time?

In this study, we use GFT to identify central variables for compliance in the context of the COVID‐19 crisis and formulate hypotheses that are subsequently tested using data collected in the first months of the COVID‐19 crisis in Flanders (Belgium). These variables relate to the three overarching goals in GFT: the *normative* goal, with variables pro‐socialness, trust in government, observed respect for rules and rule legitimacy; the *hedonic* goal, with variables fear and opportunities for fun and happiness; and the *gain* goal, with variable worsened income position. Based on GFT, we were also able to include a hypothesis on the temporal dynamics of rule compliance, that is, how the goal constellations change over time.

We test our hypotheses with data collected at three different moments during the first months of the COVID‐19 crisis in Flanders (Survey Wave 1, April 7; Survey Wave 2, April 28; Survey Wave 3, June 30). Wave 1 took place at the peak of infections, with a stringent lockdown, as at the time it was not yet known whether the measures were working. Wave 2 took place when it was clear that the lockdown was working as the infections and hospitalizations were declining and the future relaxation of measures was announced. Finally, Wave 3 occurred when the infections were at a comparatively very low point and further suspension to the remaining lockdown measures would take effect the very next day. To analyze our data, we use regressions with bootstrapping. We demonstrate that GFT can explain the dynamics of the drivers of rule compliance during a prolonged crisis like the COVID‐19 pandemic. We discuss how this study further refines and strengthens our theoretical understanding of the drivers of compliance and their contingencies and conclude with suggestions for further research and policy implications.

## 2 Rule compliance

Rule compliance occurs when regulatees do what the regulations stipulate that they should do (OECD 2014). In general, regulatees can be individual citizens that are required to comply with, for example, tax rules (Alleyne & Harris 2017) or traffic rules. Or they can be organizations that need to comply with regulations regarding, for example, food safety regulations (Bradford‐Knox & Neighbour 2017) or quality of health care regulations (Seddon & Currie 2013). In the present study, we look at individual citizens as regulatees and the government as regulator.

Compliance can be primarily enforced using sanctions or be obligation‐based. Obligation‐based compliance is more voluntary (Lindenberg 2001): people feel the obligation to comply with the rules because it is important for the greater collective; irrespective of the chances of getting caught (Tyler 2006). Compliance often takes an effort though and may go against people's self‐interest. In this study, we look at compliance with the behavioral rules that help prevent the spread of the coronavirus (such as keeping distance). This takes considerable effort from citizens and, in many cases, leads to loss of income.

## 3 Goal Framing Theory

GFT acknowledges the existence of different decision frames that guide people's decisionmaking and, hence, the motivational dynamics of one's behavior. The frames are based on three overarching goals: hedonic (“to feel good right now”), gain (“to preserve and improve resources”), and normative (“to act appropriately”). The most salient goal frames the decisionmaking and behavior, but the other two goals influence that salience from the background: the more compatible they are, the more they strengthen the salience of the dominant goal; and when they are incompatible, they weaken the salience (Lindenberg 2006, 2017; Lindenberg & Steg 2007). Without additional supports for these goals, there is a natural pecking order: the hedonic goal is the strongest, and the normative goal the weakest. This implies that the normative goal must have additional strong supports to become, and remain, the most salient goal.

For sustainable rule compliance, the normative goal needs to be most salient, because that goal relates to a feeling of obligation to comply with rules. When the gain goal or the hedonic goal is most salient, then rule compliance is usually unstable (Tenbrunsel & Messick 1999; Lindenberg *et al*. 2021). Rule compliance with a dominant gain goal will only work in high sanction and enforcement regimes with high chances of getting caught. Rule compliance with a dominant hedonic goal is only likely when it makes one feel good right now or avoids feeling bad. Acting appropriately refers to complying with the norms of the social groups that one identifies with. It implies that you are willing to consider the needs of others in these groups even if that goes against your own self‐interest (as in the gain goal) or instant gratification (as in the hedonic goal). Formal rules, however, are not always seen as social norms and are, thus, not automatically covered by the normative goal. This is a central step in GFT's theory on rule compliance: how to make formal rules be seen as social norms so that the normative goal applies to them as well. Lindenberg (2017) claims that legitimacy is the major link between the rules imposed by institutions and the social norms that are part of the normative goal.

In sum, there are two main influences on obligation‐based compliance. First, those influences that make rules being covered by the normative goal (legitimization process of rules) and second, those influences that increase or decrease the salience of the normative goal from the cognitive background (the (in)compatibility of gain and hedonic goals).

## 4 Rule legitimization processes: From rules to social norms

Rule compliance is argued to be related to individual differences in pro‐socialness. In functioning democracies, government rules are legitimate at least to some degree and, therefore, people who are more pro‐social are more likely to have a more salient normative goal and, thus, are more likely to comply with rules. Pro‐socialness is a person's proneness to take the interests of others and the collective into account, even if it presents a disadvantage for themselves (Bogaert *et al*. 2008; Boone *et al*. 2010; Declerck *et al*. 2013). People who are more pro‐social are more sensitive to complying with social norms and, thus, chronically activate their normative goal. Hence, we hypothesize:

Hypothesis 1 Citizens' pro‐socialness has a positive effect on rule compliance.

There is empirical support for this in the COVID‐19 crisis context. American adolescents who reported higher self‐interest values (in other words were low on pro‐socialness) were less likely to observe physical distancing rules (Oosterhoff & Palmer 2020). And studies with adults found that inducing empathy (Pfattheicher *et al*. 2020) and altruism (Brooks *et al*. 2020) in the public elicited higher motivation to comply with the distancing measures.

Even though individual differences in pro‐socialness are important, GFT suggests that social factors affecting the legitimization of rules are even more important. Legitimization of rules, that is the degree to which these rules are seen as norms, is likely to vary according to the three conditions specified by GFT (Lindenberg *et al*. 2021): the legitimacy of the rule‐making authority, the rules make sense, and the perceived support for the rules.

### 4.1 Legitimacy of rules based on legitimate authority: Trust in government

Lindenberg *et al*. (2021) argue that for the legitimization process, in which rules turn into social norms, the rule‐making authority needs to be seen as legitimate. The legitimacy of the rule‐making authority has been shown to be important in other theories. People are more likely to obey the law when they judge that the rule‐making authorities act with integrity and will treat them fairly (Tyler 2006). Institutional integrity – which involves fairness, trust, and rule‐making authority's lived commitment to principles in its mission – helps to motivate compliance (Braithwaite 2009). Lindenberg *et al*. furthermore stress that the rule‐making authority needs “to be seen to be treating their own rule as a norm by not showing disrespect for it” (Lindenberg *et al*. 2021, p. 432). This phenomenon is also seen in the ethical leadership literature; setting the example in the positive sense is important for ethical leaders but violating one's own norms has a more vicious impact (e.g. Brown & Treviño 2006).

We argue that trust in the rule‐making authority is a concept that is very closely related to this concept of legitimate authority in GFT. Authority can be trusted when it is competent, benevolent (takes other people's interests into account, even if it goes against their own interests) and acts with integrity (adheres to its own principles that people find acceptable) (Grimmelikhuijsen and Knies, 2017). Treating people fairly links to the benevolence dimension of trust, and adhering to one's own principles and rules is part of the integrity dimension. In other words, we expect that individuals with higher levels of trust in government will be more likely to comply with government measures.

Hypothesis 2 Citizen trust in government has a positive influence on rule compliance.

There is also convincing empirical evidence that, under normal conditions, there is a positive relationship between trust and rule compliance. Low trust leads to low compliance, and high trust leads to high compliance (e.g. Ayres & Braithwaite 1992; Braithwaite & Makkai 1994; Murphy 2004; Six & Verhoest 2017). Braithwaite and Makkai (1994, p. 2) assert that if those who are being regulated are treated as trustworthy, “they will repay this respect with voluntary compliance” (see also Feld & Frey 2002). This will only happen when there is sufficient reciprocal trust between regulators and those regulated (Korsgaard 2018). Empirical evidence furthermore suggests that trust plays an important role in the acceptance of and compliance to state regulations (Braithwaite & Makkai 1994; Tyler 2006) and that government officials who act in a trustworthy manner have higher chances of eliciting compliance (Levi & Stoker 2000). In sum, many studies show that, in normal times, trust between the regulator and the regulatee nourishes voluntary, obligation‐based compliance.

Trust in government is also an important determinant of citizen's compliance in times of crisis, especially epidemics and pandemics (e.g. Tang & Wong 2003; Rubin *et al*. 2009; Blair *et al*. 2017; Vinck *et al*. 2019). For the COVID‐19 pandemic, studies found that respondents who exhibit higher institutional trust are more likely to comply with protective behavior advice (Bargain & Ulugbek 2020; Brouard *et al*. 2020; Devine *et al*. 2020; Jørgensen *et al*. 2020).

### 4.2 Legitimacy of rules based on rules making sense

The second condition for rules to become social norms specified by Lindenberg *et al*. (2021) is that the rules make sense. In other words, they must be seen as effective and appropriate (Murphy *et al*. 2009). Weber (1978) showed that in non‐autocratic societies with democratic institutions, rules are not seen as edicts, but as enforceable means to achieve a positive result for the common good (rational‐legal legitimacy of rules). This aspect of the expected instrumentality of official rules as the basis for legitimacy has been recognized by many theories on rule compliance, most prominently in Tyler's work (Tyler 2006). Yet, this has not yet been related to the dynamics of the normative goal, as in GFT. For GFT, this aspect of legitimacy, the “making sense” of rules means that they are effective, but with an eye to the common good, meaning that they must also be appropriate (i.e. without unduly restricting citizens' freedoms). In sum, we hypothesize that a higher level of perceived rule effectiveness and perceived rule appropriateness will lead to a higher level of compliance with rules.

Hypothesis 3a Perceived rule effectiveness has a positive effect on rule compliance.

Hypothesis 3b Perceived rule appropriateness has a positive effect on rule compliance.

Empirical support for the effect of rule effectiveness on voluntary compliance during COVID‐19 crisis was found in Clark *et al*. (2020).

### 4.3 Legitimacy of rules based on observed respect for rules

For rules to be seen as norms, it is not enough that the authorities are legitimate and that the rules make sense. Regarding the question of whether one should feel obligated to follow government rules, people are also sensitive to what other people are doing. Do people observe that most others respect the rules? Observing respect for rules increases the salience of the normative goal and observing disrespect lowers the salience of this goal. Hence, the third condition for rules to become social norms is that people perceive that the rules have widespread support. Because of this effect of observing others' respect for rules on the salience of the normative goal, the very goal to comply with legitimate rules is socially contagious (Lindenberg *et al*. 2021). This is part of social contagion of rule compliance. In this study, we, thus, focus on the observed respect for rules and formulate the following hypothesis:

Hypothesis 4 Observing other people respect the rules has a positive effect on rule compliance.

Again, empirical research supports this hypothesis. Studies conducted during earlier pandemics (such as SARS and Ebola) as well as during the current COVID‐19 crisis show that individuals who comply with measures do so because of social pressure and not wanting to be seen as going against the collective commitment (Cava *et al*. 2005; Desclaux *et al*. 2017; Webster *et al*. 2020).

The above hypotheses address how to strengthen the salience of the dominant normative goal and how to make rules into social norms. We now turn to the role of the other two goals in the background.

## 5 Compatibility of background goals

The most salient goal frames the decisionmaking and the other two goals influence that salience from the background. The more compatible they are with the dominant goal, the more salient the dominant goal and the more incompatible they are, the more they weaken the dominant goal. Because sustained rule compliance requires a dominant normative goal, the hedonic and gain goals are in the background.

### 5.1 Compatibility of hedonic goal

To what degree is the hedonic background goal compatible with the dominant normative goal? Wanting to experience pleasure is usually incompatible with rule compliance since it implies avoiding making much effort. It, therefore, makes the hedonic goal less compatible with the normative goal. Avoiding or reducing fear, especially in a crisis such as the COVID‐19, is likely to support the normative goal from the background, even if compliance takes an effort. This fear has two dimensions. First, the fear related to the severity of the risk of contracting COVID‐19: when you get infected you may get seriously ill. Second, the fear of contamination due to risk proximity: when the virus is nearby, there is a high chance of getting infected. In line with this, we hypothesize that the fear of COVID‐19 will lead to higher compliance with the COVID‐19 measures.

Hypothesis 5a Citizens' fear of the severity of the COVID‐19 illness (risk severity) has a positive effect on rule compliance.

Hypothesis 5b Citizens' fear of the proximity of the Coronavirus (risk proximity) has a positive effect on rule compliance.

Many empirical studies into epidemics and pandemics support this hypothesis: functional fear is a significant predictor of public health compliance (e.g. Chang *et al*. 2004; Robertson *et al*. 2004; Chew *et al*. 2020; Harper *et al*. 2020; Raude *et al*. 2020; Zettler *et al*. 2020).

When people have many opportunities to experience pleasure and happiness, the salience of the hedonic goal is likely to be strengthened in a positive manner. The lockdown measures that were imposed to stop the spread of the coronavirus have overall reduced the opportunities for such positive experiences. The degree to which citizens can still experience pleasure and happiness has a positive effect on compliance. We, therefore, hypothesize that

Hypothesis 6 Citizens' opportunities to experience pleasure and happiness have a positive effect on rule compliance.

### 5.2 Compatibility of gain goal

The gain goal in the background is compatible with the dominant normative goal when its goal to preserve or increase one's resources can be met. In the COVID‐19 crisis, for most people, the focus is on preserving one's resources and where possible avoiding loss of resources. The more people experience loss of income due to COVID‐19 measures, the more the gain goal is incompatible with the dominant normative goal, which in turn will have a negative effect on rule compliance. So our final hypothesis is,

Hypothesis 7 A worsened income position has a negative effect on rule compliance.

### 5.3 Goal constellations over time

Because of the inherent tendency for the normative goal to decay over time relative to the hedonic and gain goals, the legitimization process of the normative goal needs to be ongoing (Lindenberg *et al*. 2021). This can be achieved in different ways. First, based on the three different bases for rule legitimization that we have identified: legitimate authority (trust in government), rules that make sense (rule effectiveness and rule appropriateness), and observed respect for rules. As the context changes, the degree to which these bases strengthen the salience of the normative goal may change as well. Second, the compatibility of the hedonic and gain goals in the background requires ongoing attention. This means that government measures need to be constantly adapted to ensure that (i) each legitimization base is as strong as possible to make the salience of the normative goal strong enough to be the dominant goal relative to the gain and hedonic goal; and (ii) the gain and hedonic goals are as compatible as possible with the normative goal.

Thus, applied to our case, as the context of the COVID‐19 pandemic changes, government measures have to adapt to continuously support the legitimization process by responding to changes in the observed respect for rules; to changes in the perceived appropriateness or effectiveness of rules; or to changes to trust in government. Also, government measures need to take into account changes in the compatibility of the background goals. How exactly this evolves is also dependent on the context to predict in a detailed hypothesis. Therefore, the hypothesis about the temporal dynamics is formulated relatively open and we explore how exactly the goal constellations change over time across the survey waves. We predict that the stronger the ongoing process of adaptation of government measures, the stronger rule compliance remains over time or, in other words, the more it is sustainable.

Hypothesis 8 A strong and continuous adaptation of government measures aimed at strengthening the salience of the normative goal, via the legitimization process and the process of enhancing the compatibility of the background goals, has a positive effect on the sustainability of rule compliance.

## 6 Method

**Figure 1:**
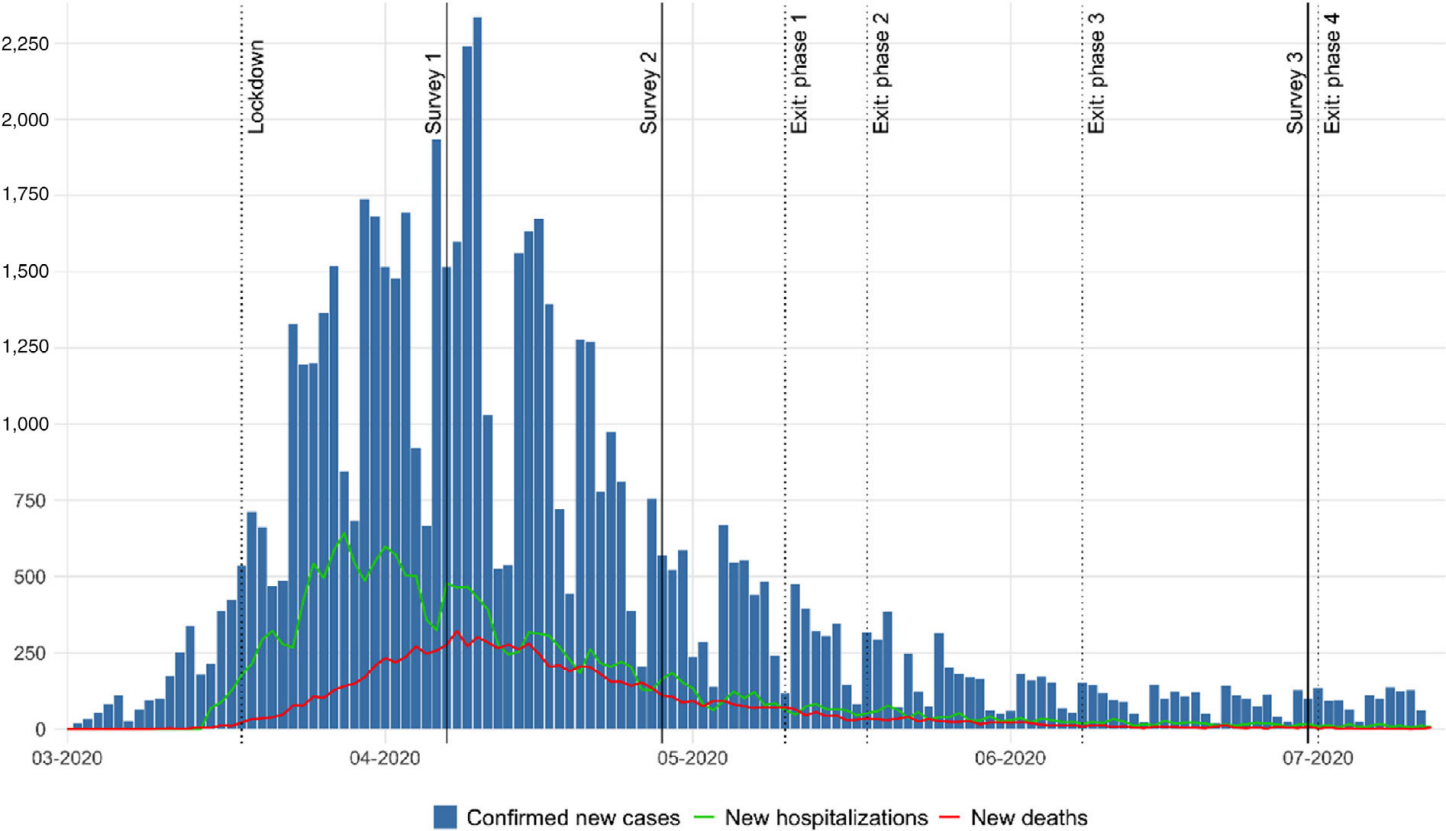
Overview of COVID‐Belgium with the most important government policies and survey dates.

The University of Antwerp conducted the Great Corona Study (GCS), a weekly survey to estimate the impact of the corona crisis. On 7 April, 28 April, and 30 June 2020, several questions on citizens' trust and compliance were included. As illustrated by Figure 1, the three surveys were set in very different contexts. While the first and second survey waves both took place during the lockdown, the epidemiological situation and perception differed (Sciensano 2020). In the first Survey Wave (*N* = 207,304) daily confirmed cases increased and some intensive care units reached maximum capacity. The number of deaths was increasing daily. It was unclear when the peak of infections would be reached. The second Survey Wave (*N* = 108,415) is situated in a period where daily COVID‐19 infection numbers declined. Furthermore, the exit‐strategy out of the lockdown was determined and communicated. Finally, Survey Wave 3 (*N* = 25,241) occurred when cases, hospitalizations, and deaths had been low for a while and major relaxation had already taken effect. One day after the survey, a fourth round of already announced relaxations of measures would enter into force (Belgian Federal Public Service 2020).

The GCS was communicated widely through news media and social media asking Belgian citizens to participate and, hence, relied on self‐selection. Participants were not randomly selected to participate in the survey. As a result, respondents are not a correct reflection of society. Belgium is a country with different language‐based communities. The survey was completed more in Flanders (Dutch‐speaking part of the country), than in the other parts of the country. In order to avoid the misrepresentation of other communities, we limit the analysis to respondents living in Flanders based on the respondents' postcode. In order to deal with issues of under‐representation, we weighted the data sample. For example, the study was completed more often by women, highly educated people, and people under the age of 65. In addition, the province of Antwerp is overrepresented. We have weighed the data by age, gender, level of education, and province. Working with weights also means that the composition of each survey wave is relatively similar (see Appendix S1 for more detailed information). The weighting factors were limited to a maximum of three.

Working with self‐reported data collected through the same questionnaire makes the results potentially sensitive to common method bias (CMB). Two additional steps were taken to reduce the likelihood of CMB. First, we avoided complex, ambiguous, or abstract items (Meier & O'Toole 2012; Podsakoff *et al*. 2012; Jakobsen & Jensen 2015). The items measuring different variables refer to focused items and concrete practices which measure current states (see Jakobsen & Jensen 2015, p. 17). Second, as visible in the specific wording of the independent variable and items measuring the dependent variables, we are measuring different issues, which are consequently based on different questions. These respective sets of questions were placed in different locations of the survey and appeared as separate webpages, not in consecutive order. A separation in the survey between independent and dependent variables measures has been suggested as a way to reduce SMB (Jakobsen & Jensen 2015, p. 17; Podsakoff *et al*. 2012). Considering the abovementioned reasons, we believe the risk of CMB to be limited. As we cannot completely rule this possibility out, results should consequently be interpreted with some care.

Overall, the measures used in this paper are based on measures used in other studies. We developed the measures in the first weeks of the lockdown by looking at literature, existing cross‐country surveys and by linking them up with new survey initiatives which were launched in other countries (e.g. the Netherlands, the UK) during the second half of March 2020. An important source for our measure development was the COVID‐19 risk perception survey of Cambridge University Winton Centre for Risk and Evidence Communication, which was conducted in 10 countries in Spring 2020 (published in Dryhurst *et al*. 2020; Schneider *et al*. 2021).

As shown in Table 1, the dependent variable “rule compliance” is measured with one item, designed in such a way to allow for changing guidelines over time, which enables us to have the same question asked in each survey wave. A robustness check was performed with a multi‐item measurement, referring to some specific measure for the second survey wave, which yielded very similar results (Appendix S1). Furthermore, as the dependent variable “rule compliance” might be also considered as an ordinal variable, the same appendix also shows the ordered logit models of the basics models discussed in Table 3, which resulted in similar findings (except that some socio‐demographic control variables turned significant). Given that the ordered logit models give similar results, in this paper, we opt to present the OLS analyses because they are easy to interpret.

**Table 1:** Variables included in regression models

	Name of variable	Question in survey	Scale
Dependent variable	Compliance	To what extent did you change your behavior in public spaces and shops according to the new guidelines?	0 (Not at all) to 10 (to a very high extent)
Control variables	Age	What is your age (in years)	—
	Female	What gender do you identify with?	1—Men 2—Female. Analyzed as dummy variables with “men” as reference
	Alone	Do you live alone, or do you have housemates?	1—I live alone 2—I have housemates. Analyzed as dummy variables with “I have housemates” as reference
	Education	What is your highest level of schooling? (If you are still a student, please answer with the level you are currently obtaining).	1—Primary education 2—Secondary education 3—Bachelor‐level education 4—Masters/PhD‐level education
Independent Variables	Pro‐socialness	To what extent do you think it is important to do things for the benefit of others and for society, even if that has disadvantages for yourself?	1 (Not important at all) to 8 (very important)
	Trust in Government	How much do you trust the federal government to tackle the COVID‐19 crisis in a good way?	1 (Do not trust at all) to 7 (trust completely)
	Rule effectiveness	To what extent do you think that the measures taken so far by the Belgian authorities have been effective in tackling the COVID‐19 crisis in general?	1 (Not at all) to 7 (to a very high extent)
	Rule appropriateness	To what extent do you think that the government measures taken in the context of the COVID‐19 crisis in Belgium put too many restrictions on people and society?	1 (Not at all) to 7 (to a very high extent)^†^
	Observed respect for rules	To what extent did people in your surroundings change their behavior in public spaces and shops according to the new guidelines?	0 (Not at all) to 10 (to a very high extent)
	Fear: risk severity	To what extent do you think being ill with the coronavirus can be serious?	1 (Not at all) to 7 (to a very high extent)
	Fear: risk proximity	To what extent do you think the risk is high that you, your family or friends will catch the coronavirus?	1 (Not at all) to 7 (to a very high extent)
	Pleasure and happiness^‡^	Have you been able to enjoy your normal, everyday activities in the past week? Have you felt reasonably happy in the last week, all things considered?	1—More than usual 2—As much as usual 3—A little less than usual 4—Much less than usual
	Worsened income position	Has your employment situation changed since COVID‐19?	1—No, not worsened because of no change or a new job 2—Yes, worsened because of technical unemployment, unemployment, or closing of own shop/company. Analyzed as dummy variables with “men” as reference

^†^ Original question is about restrictiveness; we reversed the scale to match the term “appropriateness.”

^‡^ The analyses used the mean of the two questions.

With respect to independent variables, most of them are based on previously used measures. For trust in government a one‐item measurement was used that is similar to the single‐item measurements of trust used in large cross‐country surveys, such as the European Social Survey and the European Quality of Life Survey, and that has been evaluated for their validity by the OECD in their guidelines for measuring trust (2017). A validity check was done at a later moment showing that the one item measurement correlates very highly with three items on ability, benevolence, and integrity as dimensions of trust in government. The items covering that the rules make sense – rule effectiveness and rule appropriateness – are based on similar items asked in the Cambridge University Winton Centre for Risk and Evidence Communication in their COVID‐19 multi‐country survey and the University of Twente survey conducted in the Netherlands and Poland (Proszowska 2020; Proszowska *et al*. 2020).

For the two fear measures – risk severity and risk proximity – we draw upon the questionnaires by Wise *et al*. 2020, the Cambridge University Winton Center survey (2020, published in Dryhurst *et al*. 2020; Schneider *et al*. 2021) and the survey used by the University of Twente (Proszowska *et al*. 2020). In building our measure for observed respect for rules (social contagion), we drew inspirations from the research of Lindenberg *et al*. (2021) but, since their empirical work is based on experiments, we could not copy items from those studies. We designed our question to take a specific COVID‐19 context into account, where social norms refer to the government measures to stop the spread of the virus and the behavior of others refers to observing compliance with such measures among other people. Measures on pleasure and happiness, and income were taken from the existing health part of the GCS.

Because the number of respondents differed substantially between the survey waves, the data was analyzed using the bootstrapping technique. Bootstrapping is a nonparametric approach to statistical inference based on building a sampling distribution for a statistic by resampling multiple times from the original data (Davison & Hinkley 1997). In this case, we drew 10,000 samples of 10,000 observations each. Each sample was constructed without replacement, meaning that observation cannot be used multiple times in the same sample. For every sample, weights were calculated, and linear regressions were performed. In a final step, the averages were taken for each regression value. Using the same size for all three waves has the added benefit of eliminating significance based on large samples.

## 7 Results

Table 2 reports the bootstrapped averages and standard deviation of the independent and dependent variables for each survey wave. This table provides insights into how variables evolved over time. The independent sample *t*‐tests between waves report statically significant changes: only compliance was not significant between waves 1 and 2, while only pro‐specialness was not significant between waves 2 and 3.

**Table 2:** Weighted independent samples *t*‐test for each ordinal variable between waves

	Survey Wave 1		*t*‐test (1–2)		Survey Wave 2		*t*‐test (2–3)		Survey Wave 3	
	M	SD	*t*	*P*	M	SD	*t*	*P*	M	SD
Pro‐socialness	5.95	1.50	7.46	0.000	5.80	1.45	−2.50	0.058	5.85	1.49
Trust in government	5.10	1.59	37.45	0.000	4.26	1.65	17.19	0.000	3.86	1.62
Rule effectiveness	5.55	1.35	12.47	0.000	5.27	1.40	31.24	0.000	4.63	1.47
Rule appropriateness	5.73	1.77	29.95	0.000	4.83	1.86	−24.32	0.000	5.44	1.65
Observed respect for rules	8.15	1.71	12.08	0.000	7.87	1.68	69.84	0.000	6.09	1.9
Fear: risk severity	6.53	0.98	4.40	0.001	6.45	1.02	−7.62	0.000	6.56	0.96
Fear: risk proximity	4.97	1.56	23.66	0.000	4.34	1.58	10.24	0.000	4.11	1.52
Pleasure and happiness	2.29	0.64	−9.62	0.000	2.38	0.67	21.93	0.000	2.19	0.52
Compliance	9.14	1.29	1.10	0.345	9.12	1.11	37.12	0.000	8.44	1.43

While pro‐socialness decreases significantly between the first and second waves, the means do not change much, meaning that this variable remains fairly consistent over time. On the other hand, trust in government declines substantially over time. The perceived effectiveness of government measures tackling COVID‐19 decreases as well, especially between the second and third waves. The new policies are seen as very appropriate in the first survey wave, three weeks later the evaluation is different as more people consider the measures as too restrictive. Once most restrictions are loosened, the perceived appropriateness significantly rises again. Observed respect for rules declines particularly between the second and third survey wave. Furthermore, respondents estimate the risk of catching the virus (i.e. risk proximity) significantly lower each survey wave. However, throughout all the survey waves, people are strongly convinced that being infected with the coronavirus (i.e. risk severity) could be serious. Citizens' opportunities to experience pleasure and happiness significantly increased after the first survey wave but decreased to its lowest point in survey wave three. Multicollinearity was tested for but is not an issue.

The standardized regression results are reported in Table 3. While we added the independent variables in separate models, we only show the final models with all variables mentioned above. A more detailed overview of each model can be found in Appendix S1. We also performed ordered logit regressions as robustness checks which result in similar findings^1^ (see Appendix S1).

**Table 3:** Final weighted regression result (standardized coefficients) in bootstrapping analysis without replacement for each survey wave

	Wave 1	Wave 2	Wave 3
Age	0.115***	0.120***	0.200***
Female	0.066**	0.084***	0.119***
Alone	−0.029	−0.035	0.022
Education	0.007	−0.001	−0.024
Pro‐socialness	0.033	0.024	0.093***
Trust in government	−0.005	−0.043	−0.077*
Rule effectiveness	0.024	0.054*	−0.008
Rule appropriateness	0.120***	0.125***	0.203***
Observed respect for rules	0.327***	0.309***	0.282***
Fear: risk severity	0.108***	0.136***	0.145***
Fear: risk proximity	0.030	0.044*	0.105***
Pleasure and happiness	0.040	0.034	0.016
Worsened income position	0.018	0.021	0.011
*R* ^2^	0.187	0.183	0.233
Adjusted *R* ^2^	0.184	0.181	0.230

Level of significance:

^***^ *P* < 0.001;

^**^ *P* < 0.010;

^*^ *P* < 0.050, *P* < 0.100.

Sample sizes = 10,000, number of resampling = 10,000. Shown results are averages over all resamples.

The results for each hypothesis are summarized in Table 4. Only H3b, H4, and H5a are supported throughout all three waves. Rule appropriateness (H3b), observed respect for rules (H4) and risk severity of COVID‐19 illness (H5a) are significant throughout all waves in line with expectations. The betas of observed respect for rules go down over time, but the betas for the other two variables go up over time. Two hypotheses on the background goals (H6 and H7) are never supported, indicating that pleasure and happiness, and worsened income do not have a significant effect on rule compliance. The other four hypotheses show a more varying result over time. Pro‐socialness is only significant in Wave 3, but not in the earlier waves. H1 is, therefore, only supported in Wave 3. Trust in government only becomes significant in Wave 3, but with a negative effect. H2 is, therefore, rejected in Wave 3 and not supported in Waves 1 and 2. Rule effectiveness, one of the items measuring whether the rules make sense is only significant in Wave 2, making H3a supported only in Wave 2, and not in the other waves. Finally, risk proximity is significant in Waves 2 and 3 with an increasing beta coefficient, but not in the first Wave, implying that H5b is supported in Waves 2 and 3, but not in Wave 1.

**Table 4:** Summary of results for each hypothesis for each wave

	Wave 1	Wave 2	Wave 3
H1: Pro‐socialness (positive effect)	Not supported	Not supported	Supported
H2: Trust in government (positive effect)	Not supported	Not supported^†^	Rejected
H3a: Rule effectiveness (positive effect)	Not supported	Supported	Not supported
H3b: Rule appropriateness (positive effect)	Supported	Supported	Supported
H4: Observing other people respect the rules (positive effect)	Supported	Supported	Supported
H5a: Fear of the severity of COVID‐19 illness (risk severity – positive effect)	Supported	Supported	Supported
H5b: Fear of the proximity of the Coronavirus (risk proximity – positive effect)	Not supported	Supported	Supported
H6: Opportunities to experience pleasure and happiness (positive effect)	Not supported	Not supported	Not supported
H7: A worsened income position (negative effect)	Not supported	Not supported	Not supported
H8: Continuous adaptation of measures (positive effect)	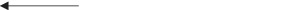	Supported	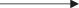

^†^ See Endnote 1. H2 is already rejected in Wave 2 according to the ordered logit models.

H8 looks at the dynamics over time. We see that rule compliance remains as high in Wave 2 as it did in Wave 1, so the legitimization process that drives rule compliance remains sufficiently strong. In Wave 3, however, we see a significant decline of rule compliance, suggesting that the process of legitimization is no longer strong enough to sustain the high rule compliance. Overall though, rule compliance remains high (8.44 on a scale from 0 to 10).

## 8 Discussion

We discuss the first seven hypotheses that were tested relatively briefly, because it is theoretically and societally more relevant to discuss Hypothesis 8 and the temporal dynamics over time. We do this in the second section by interpreting the results for the whole constellation of goals and context factors for each wave. In the third section, we reflect on the limitations of the study.

### 8.1 Discussion of hypotheses 1–7

The results for pro‐socialness (H1) and trust in government (H2) are interesting as they are not in line with many other regression analysis‐based studies, even the recent ones on COVID‐19. In these studies, pro‐socialness is found to have a significant effect on compliance (; Oosterhoff & Palmer 2020; Pfattheicher *et al*. 2020). In our study, it had a significant effect in Waves 1 and 2, until the observed respect for rules was added in the regression analysis (see Appendix S1). This suggests that observing respect for rules among others can get even people low on pro‐socialness to comply in Waves 1 and 2, but not anymore in Wave 3. Over the three waves, the mean of this variable stays more or less the same, which is in line with previous research that shows that pro‐socialness is a personal characteristic that remains quite stable over time (Bogaert *et al*. 2008). The fact that H1 is only supported for Wave 3 might indicate that as risk proximity and observed respect for rules decline, personality factors relating to rule compliance become more important.

Interestingly, trust in government (H2) is not significant in the full models of Waves 1 and 2, which is unusual; it even has a significant negative effect on compliance in Wave 3, which is unprecedented (Tang & Wong 2003; Rubin *et al*. 2009; Blair *et al*. 2017; Vinck *et al*. 2019; Bargain & Ulugbek 2020). The perceived effectiveness of the rules plays a role in explaining these results, as does the observed respect for rules. In Waves 1 and 2, trust in government starts as significant and positive when added to the model but loses that significance once we add rule effectiveness. In Wave 2, the addition of observed respect for rules gives trust in government a significant negative effect, but this significance disappears as variables related to the background goals are added. In Wave 3, trust starts as insignificant and adding effectiveness of rules to the model gives trust a significant negative effect. Next, adding the observed respect for rules almost doubles this effect. Strikingly, when re‐running the analyses without rule effectiveness, the trust variable still shows the same outcomes in the full models. Moreover, we tested for interaction effects between trust in government on the one hand and rule effectiveness and observed rules on the other, but these interaction terms did not yield any significant effects, nor did they change the outcomes of the trust variable or the other variables.

The two hypotheses for rules making sense (H3a and H3b) have been studied in other research and the results are largely in line with expectations (Tyler 2006; Murphy *et al*. 2009). For H3a (effectiveness), our results show that the rules making sense matters in most waves, and effectiveness of rules only becomes insignificant when observed respect for rules is added to the model in Waves 1 and 3. This latter variable is a new variable that has, at least to our knowledge, not yet been studied in previous research on rule effectiveness and rule appropriateness, which may explain the small deviation from expectations.

Observed respect for rules (H4) is important for rule compliance in all three waves. This variable, shown here to be quite central to rule compliance, is not often part of other studies and could explain the divergence of results. Despite its theoretically central role in GFT, GFT is a relatively recent theory in regulation research (Etienne 2013; Lindenberg *et al*. 2021), which could explain the absence of this social contagion variable in most other regulatory compliance studies. Also, in much of the other regulation research, the context is such that it is difficult to observe whether other regulatees observe the rules. For example, it is difficult to know for a mine worker whether in other mines rules for workers' health and safety are complied with (e.g. Gunningham & Sinclair 2009), or for taxpayers whether other people pay their taxes (e.g. Murphy 2004).

That fear of COVID‐19 is an important factor driving compliance (H5a and H5b) has been confirmed in many studies on pandemics (e.g. Harper *et al*. 2020; Wise *et al*. 2020; Zettler *et al*. 2020). Risk severity – referring to the seriousness of the COVID‐19 illness – stimulates rule compliance in a consistent way across waves. The fact that in our study risk proximity (H5b) is not significant in Wave 1 is most likely due to not many people knowing someone who was seriously ill with COVID‐19. The insignificance of the opportunities for pleasure and happiness (H6) and worsened income position (H7) throughout all the waves was only partially unexpected, since the last Wave was launched only three to four months after the first lockdown was introduced. These variables may become significant during the second lockdown in the autumn/winter of 2020/2021, as restrictions are then likely to impose a larger toll on the enjoyment of activities and on one's income position.

Two control variables are significant throughout the waves: age and gender. Females and older people are more law‐abiding. Age and gender are indicators of the chronic activation of the normative goal: older people are more likely to act appropriately than younger people (Velez & Lopez 2013; Matsumoto *et al*. 2016) and the same holds for women compared to men (Croson & Gneezy 2009; Choy *et al*. 2017).

### 8.2 Hypothesis 8: Interpretation of results in context of crisis over time

Given GFT's contingent nature with context factors impacting the constellation of the goal frame, the best test of the theory is to look at the total picture of each wave. Hypothesis 8 addresses the temporal dynamics of the context factors impacting on rule compliance. To what extent were the government measures continuously adapted for rule compliance to be sustainable? In this section, we first look at the total picture for each wave and how that changes over time. The COVID‐19 crisis is unique because governments imposed strict measures that were unprecedented in peacetime democracies. The government measures reduced citizens' freedoms and impacted incomes and well‐being of many, impacting on the hedonic and gain goals. For the hedonic goal, the reduced opportunities for pleasure and happiness make the goal incompatible with the normative goal, while the fear of the virus makes it compatible. The results show that on balance, the hedonic goal is compatible with the normative goal, because the fear of COVID‐19's severity – which is compatible with the normative goal – is significant in all waves and the opportunities for happiness and pleasure variable – which is incompatible – is never significant. The variable worsened income position that measures the gain goal is never significant, suggesting that the gain goal had no significant effect on the goal constellation.

Also, the rule legitimizing processes worked to strengthen the salience of the normative goal, in particular the observed respect for rules (social contagion) and the appropriateness of the rules. Three factors – fear (severity), observed respect for rules, and rule appropriateness – drove compliance through all the waves. As the crisis evolved and the measures worked in the sense that the prevalence of the virus was drastically reduced and hospitalizations and deaths came down, more distinct groups with different drivers for (non)compliance emerged.

The picture that our analyses paint for *Survey Wave 1* is the following. At this point during the crisis (7 April 2020), it is yet unclear whether the spread of the virus can be controlled, because the rate of infections, hospitalizations, and deaths is still increasing rapidly. There is also no real treatment for those infected and hospitalized. In this context, compliance is mainly predicted by observed respect for rules, fear of COVID‐19 (risk severity), and rule appropriateness. The normative goal is dominant and the hedonic variable fear of COVID‐19 is strongly present in the background as compatible with the normative goal, both supporting compliance. The gain goal does not play a role. Of the control variables, age and gender are significant. The full model explains 18.5 percent of rule compliance with observed respect for rules being the independent variable with the highest explanatory power.

In this phase of the crisis, compliance with the national lockdown measures is very high, leading to empty streets and people staying at home. It is very clear and easy to observe that most other people respect the rules. Fear of the virus is high and perceptions of risk severity (as doctors and nurses are unsure how to treat patients) help to foster compliance. As the virus is still an unknown entity, the social distancing rules make sense. Government restrictions on individual freedoms are considered to be appropriate and non‐pharmaceutical interventions seem to be the only toolkit available (Flaxman *et al*. 2020).

Interestingly, pro‐socialness, trust in government, and effectiveness of rules do not impact compliance significantly in this phase of the crisis. For the first two variables, that is surprising given extant theory (see above). Apparently, citizens' fear of the virus and observed respect for rules have such a strong impact on the normative goal that pro‐socialness becomes irrelevant. In this phase, it also makes sense for self‐centered people to comply. In this study, it seems not to matter whether people trust the government or not: when fear is that high (high compatibility of hedonic goal), observed respect for rules and appropriate rules are enough to turn the rules into social norms (high salience of the normative goal). Even though it takes a lot of effort to comply with these rules, the fear is high enough to compensate for this, making the hedonic goal compatible in the background (Lindenberg *et al*. 2021).

In sum, compliance in Wave 1 seems to be driven by a strong dominant normative goal, which, in turn, is driven by the effect of legitimate rules that are seen as social norms, with strong support from the hedonic background goal due to the fear of the virus.

By the time of *Survey Wave 2* (28 April 2020), the lockdown is still ongoing, and measures are still as stringent, but it has become clear that the epidemiological measures have worked to contain the spread of the virus: the number of new infections, hospitalizations, and deaths has come down substantially. Also, the first relaxations have been announced. In this context, rule compliance is as high as it was on April 7 (no significant change, see Table 2) and is positively predicted by observed respect of the rules, fear of COVID‐19 (both risk severity and risk proximity), rules that make sense (both appropriateness and effectiveness of measures) and age and gender are significant control variables. The full model explains 18.2 percent of rule compliance.

All variables that reflect the normative goal except for pro‐socialness and trust in government are significant in this Wave. Rule effectiveness goes down significantly as more people see the rules as less effective. This makes the variable significant in the regression model, as those who still see the rules as effective will more likely comply, compared to those who see the rules as less effective. However, just as in Wave 1, observed respect for rules has the strongest explanatory power and hence still matters most for explaining compliance.

Both variables measuring the fear dimension in the hedonic goal are significant with a positive effect on compliance and compared to Wave 1 their values go down (Table 2). As the prevalence of the virus in society is lower than in Wave 1, risk proximity becomes significant, with those who see the risk of contamination as still high, complying more. Both beta coefficients go up and the explanatory power that the fear items add to the model is higher than in Wave 1. The pleasure and happiness dimension of the hedonic goal and the gain goal are still not significant. The results suggest that different groups of respondents start to have different motives for compliance and this trend becomes even more pronounced in Wave 3.

Finally, in *Survey Wave 3* (30 June 2020), the prevalence of the coronavirus in society is much lower than in Wave 2, some substantial lockdown measures have been lifted and further relaxation of the lockdown measures has been announced to come into force soon. In this context, rule compliance goes down significantly. In this phase, compliance is positively predicted by the observed respect for rules, fear of COVID‐19 (both risk severity and risk proximity), rule appropriateness, pro‐socialness, and age and gender. For the likely reasons explained above, trust in government has a significant negative effect on rule compliance. The full model explains 23.1 percent of rule compliance, which is the highest of all waves. Interestingly, whereas all significant changes of variables over time are downwards, rule appropriateness goes up, which may be explained by the substantial relaxation of measures that is about to happen – bringing the measures more in line with how people experience the risks of the virus (Table 2).

In Wave 3, more differentiation in groups that are more or less likely to comply starts to manifest itself. First, compliance becomes more reliant on stable personal characteristics: the beta coefficients for age and gender go up over time and pro‐socialness becomes significant. Thus, more young people are failing to comply compared to older people; more women are complying compared to men; and more self‐interested people are failing to comply compared to pro‐social people.

Secondly, as fear of contamination (risk proximity) is further reduced in Wave 3 and continuing to comply with the measures requires more and more personal effort, the support of the hedonic goal for the normative goal goes down. People are less afraid and find it harder to continue to comply, explaining the significant drop in compliance.

Thirdly and surprisingly, trust in government moves from being insignificant in the restricted model without the other independent variables, to have a significant negative effect in the full model. The cross‐tabulations show that there are low‐trustors that comply with the rules. This suggests that there is a group of rule compliers who believe that government relaxed the measures too soon, which ultimately lowered their trust in government. No prior studies of the impact of trust on compliance have found this negative effect (see e.g. systematic overview by Six & Verhoest 2017).

Overall, the picture that the data seem to be sketching is that, in Wave 3, the young are complying a lot less and men are complying less. Among those that are still complying, there are different constellations of factors influencing that compliant behavior: those that comply because of their pro‐socialness; those who believe that government is not strict enough anymore and hence do not trust government, but still comply; those that comply because of fear; and those that comply because they see others complying (in their social circles). This last group is present in all waves and, thus, important evidence for an effect of social contagion that is not often considered in studies of rule compliance in regulation research.

Looking over all three waves, the overall conclusion appears to be that, despite continuous adaptations of government measures, the voluntary basis for rule compliance becomes more fragile over time, with a more differentiated pattern of drivers of compliance emerging. This provides support for Hypothesis 8 and makes even the decline of rule compliance in line with GFT (Lindenberg *et al*. 2021).

### 8.3 Limitations of the study

The study has several limitations, which mainly relate to the data collection. First, the sample is not representative of citizens of Flanders and, by applying weights, we partly corrected for this. Since we are interested in the relationships between variables and not their absolute scores, this is less relevant. Further research should, however, use a sufficiently representative sample to correct for this potential bias. Second, we did not use panel data, so the respondents in SW2 and SW3 could be different people. The control variables across the waves did not vary much, which suggests that this may not be a big issue. Also, the fact that people needed to get the link via email or social media, suggests that to a large extent the same people were reached. Third, the study focuses only on Flanders, Belgium. We suggest that the pandemic in Flanders and Belgium is playing out in a roughly comparable manner during our time of measurement to many other European countries. The dynamics in Asian, African, and American countries appear different. Cultural differences may also lead to different degrees of pro‐socialness, trust, and legitimacy. Further research can investigate that for completeness.

Next, since our survey questions were integrated in a larger survey on health and well‐being during the COVID 19 crisis, we were forced to be very efficient in how to measure items to keep the survey sufficiently short. Several concepts are measured by single items, like trust in government, which are accepted in the literature. Moreover, with respect to the measures of two core variables, we did extra robustness checks or validity checks (i.e. for rule compliance and for trust in government). However, future research should validate the findings by using multi‐item measures for these concepts. Although trust in government and rule effectiveness are conceptually distinct, represent different variables in our theoretical model, and there was no multicollinearity, our empirical data suggest that respondents do respond to both measures in a rather similar way. Regardless, removing rule effectiveness out of the analyses did not change the results in a substantive way (see Appendix S1). Using multi‐item measures with trust in government being measured in general, rather than in relation to COVID‐19, and in relation to its dimensions (ability, benevolence, and integrity), future research may find a clearer empirical distinction between both variables. Although further analyses with interaction effects between trust in government on the one hand, and rule effectiveness, rule appropriateness, and observed respect for rules on the other hand neither yield significant effects for the interaction terms nor change the findings of our analyses, future research should explore more sophisticated causal schemes with indirect effects. Finally, future research should aim to have a more balanced measure of the gain goal, including for example the (financial) costs of rule violation.

## 9 Conclusions

This study set out to investigate what drives rule compliance during the COVID‐19 crisis. While there have been several other studies across the globe that are doing the same, we make five contributions which each call for further research. We conclude with implications for public policy.

First and foremost, we derived our hypotheses deductively from Lindenberg's GFT and in particular its theory on explaining rule compliance as formulated in Lindenberg *et al*. (2021). This study provides the first empirical test of this theory and shows that GFT is highly relevant for understanding rule compliance. Given constraints on the number of items we could put to respondents, further research should use dedicated surveys to more directly test GFT's theory on rule compliance. This can be done in a relatively static situation, as is the case in many more traditional regulatory situations, or dynamically over time, as we did.

Second, while some other studies also look across time using different waves (Lim & Nakazato 2020; van Rooij *et al*. 2020; Nivette *et al*. 2021) they usually integrate that in their regression analyses by adding a variable for the wave. We have not done that, because we are interested in the constellation of independent factors that influence compliance. In that sense, we are using a more case‐based analysis (Byrne & Ragin 2009). Further research into the dynamics of compliance over time may benefit from a case‐based rather than a variable based analysis, for example, using Qualitative Comparative Analysis (Rihoux & Ragin 2008) to further our understanding of the constellations of goals and the impact of context factors, in particular those legitimizing rules into social norms.

Third, using GFT to identify relevant factors, several more unusual factors are introduced that prove to have significant effects on compliance, like observed respect for rules (social contagion). And two other factors that are not significant in this study, but may become significant in later COVID‐19 studies during the autumn and winter of 2020/2021, are opportunities for pleasure and worsened income. This study shows that they should more often be included in rule compliance models, when the context calls for it.

Fourth, we find that the effect of trust in government on compliance is more nuanced than often claimed in the literature, especially during crises and even leading to a negative effect in Wave 3. Adding observed respect for rules to the regression analyses with trust creates this negative effect. This may be specific to the COVID‐19 crisis, but it is worth investigating whether there are other contexts where this may also be the case.

Finally, the data suggest that as the COVID‐19 crisis lasts longer, the more blanket effect of fear and observed respect for rules on compliance makes way for a more nuanced differentiation of differently motivated groups of people. The variables used to indicate how background goals of hedonism and gain are impacting rule compliance turned out to be insignificant, but we speculate that during the autumn and winter of 2020/2021 these factors may become significant in explaining compliance. Further research is needed to test that hypothesis.

### 9.1 Policy implications

This study has important implications for public policy. It shows that in democratic societies obligation‐based compliance with government rules is very context‐sensitive. When the context changes over time, as it does during the COVID‐19 crisis, citizens' motives for complying with government measures change as well. After the highly uncertain situation of the first months of the crisis, a wide variety of factors drives citizen compliance as personal and contextual circumstances vary. Public policy and government communication need to adapt to these changing contexts as well as address all the different groups of citizens.

Other implications of the study are that governments need to communicate clearly the seriousness of the risks to citizens. This may include, we speculate here, addressing (social) media messages that do not acknowledge the seriousness or even claim it is a conspiracy. Furthermore, governments should think about how to facilitate that citizens can observe others complying and respecting the rules. Media and government should not over‐emphasize or one‐sidedly highlight rule violations as these may strengthen negative social contagion, leading to an increased rule violation. And finally, the delicate balance between when and how much to tighten or relax rules is crucial. The rules should always make sense to enough people. So, governments need to be clear about the logic behind the tightening or relaxation of rules, avoiding (suggestions of) unfair lobbying.

One implication that does not directly follow from this study, but does follow from GFT, is that authority figures need to be extra careful in following the rules, because of the impact on the rule legitimating process. During the period we studied the Belgium situation, such an event did not happen, but in the United Kingdom, citizens' trust in government decreased substantially after Prime Minister Johnson refused to sack his chief adviser Cummings who had broken the rules (McKay 2021).

## Supporting information


**Appendix** **S1**. Supporting Information.

## Data Availability

Research data are not shared.

## Endnote

^1^
There are small differences, some socio‐demographic variables show a negative significant effect (the variable alone in Wave 1 and Wave 2; and the variable education in Wave 3). “Trust in government” also becomes negatively significant in Wave 2 (whereas in the OLS model it only becomes significant in Wave 3), meaning the hypothesis is rejected one wave earlier. Hence, the explanation in the discussion provided for the negative effect of trust for Wave 3 applies one wave earlier.

## References

[rego12440-bib-0001] Alleyne P , Harris T (2017) Antecedents of Taxpayers' Intentions to Engage in Tax Evasion: Evidence from Barbados. Journal of Financial Reporting and Accounting 15, 2–21.

[rego12440-bib-0002] Ayres I , Braithwaite J (1992) Responsive Regulation: Transcending the Deregulation Debate. Oxford University Press, USA.

[rego12440-bib-0003] Bargain O , Ulugbek A (2020) Poverty and Covid‐19 in Developing Countries. Bordeaux University, Bordeaux.

[rego12440-bib-0004] Becker GS (1968) Crime and Punishment: An Economic Approach. The Economic Dimensions of Crime, pp. 13–68. Springer, New York.

[rego12440-bib-0005] Belgian Federal Public Service (2020) Phase 4 of the Phasing out Will Start on 1 July | Coronavirus COVID‐19. 24 June.

[rego12440-bib-0006] Blair RA , Morse BS , Tsai LL (2017) Public Health and Public Trust: Survey Evidence from the Ebola Virus Disease Epidemic in Liberia. Social Science & Medicine 172, 89–97. 27914936 10.1016/j.socscimed.2016.11.016

[rego12440-bib-0007] Bogaert S , Boone C , Declerck C (2008) Social Value Orientation and Cooperation in Social Dilemmas: A Review and Conceptual Model. British Journal of Social Psychology 47(3), 453–480. 17915044 10.1348/014466607X244970

[rego12440-bib-0008] Boone C , Declerck C , Kiyonari T (2010) Inducing Cooperative Behavior among Proselfs Versus Prosocials: The Moderating Role of Incentives and Trust. Journal of Conflict Resolution 54(5), 799–824.

[rego12440-bib-0009] Bradford‐Knox R , Neighbour S (2017) Food Safety Compliance Approaches: Case Study of a Primary Authority Partnership between E.H. Booths Ltd and Preston City Council. British Food Journal 119(4), 744–758.

[rego12440-bib-0010] Braithwaite J , Makkai T (1994) Trust and Compliance. Policing and Society: An International Journal 4(1), 1–12.

[rego12440-bib-0011] Braithwaite V (2009) Defiance in Taxation and Governance: Resisting and Dismissing Authority in a Democracy. Edward Elgar Publishing, Cheltenham.

[rego12440-bib-0012] Braithwaite V , Murphy K , Reinhart M (2007) Taxation Threat, Motivational Postures, and Responsive Regulation. Law & Policy 29(1), 137–158.

[rego12440-bib-0013] Brooks SK , Webster RK , Smith LE *et al*. (2020) The Psychological Impact of Quarantine and How to Reduce It: Rapid Review of the Evidence. The Lancet 395(10227), 912–920. 10.1016/S0140-6736(20)30460-8 PMC7158942 32112714

[rego12440-bib-0014] Brouard S , Vasilopoulos P , Becher M (2020) Sociodemographic and Psychological Correlates of Compliance with the COVID‐19 Public Health Measures in France. Canadian Journal of Political Science 16(4), 1–6.

[rego12440-bib-0015] Brown ME , Treviño LK (2006) Ethical Leadership: A Review and Future Directions. The Leadership Quarterly 17(6), 595–616.

[rego12440-bib-0016] Byrne D , Ragin CC (2009) The Sage Handbook of Case‐Based Methods. Sage Publications, Los Angeles.

[rego12440-bib-0017] Cava MA , Fay KE , Beanlands HJ , McCay EA , Wignall R (2005) Risk Perception and Compliance with Quarantine during the SARS Outbreak. Journal of Nursing Scholarship 37(4), 343–347. 16396407 10.1111/j.1547-5069.2005.00059.x

[rego12440-bib-0018] Chang H‐J , Huang N , Lee C‐H , Hsu Y‐J , Hsieh C‐J , Chou Y‐J (2004) The Impact of the SARS Epidemic on the Utilization of Medical Services: SARS and the Fear of SARS. American Journal of Public Health 94(4), 562–564. 15054005 10.2105/ajph.94.4.562 PMC1448298

[rego12440-bib-0019] Chew QH , Wei KC , Vasoo S , Chua HC , Sim K (2020) Narrative Synthesis of Psychological and Coping Responses Towards Emerging Infectious Disease Outbreaks in the General Population: Practical Considerations for the COVID‐19 Pandemic. Tropical Journal of Pharmaceutical Research 61(7), 350–356. 10.11622/smedj.2020046 PMC7926608 32241071

[rego12440-bib-0020] Choy O , Raine A , Venables PH , Farrington DP (2017) Explaining the Gender Gap in Crime: The Role of Heart Rate. Criminology 55(2), 465–487.

[rego12440-bib-0021] Clark C , Davila A , Regis M , Kraus S (2020) Predictors of COVID‐19 Voluntary Compliance Behaviors: An International Investigation. Global Transitions 2, 76–82. 32835202 10.1016/j.glt.2020.06.003 PMC7318969

[rego12440-bib-0022] Croson R , Gneezy U (2009) Gender Differences in Preferences. Journal of Economic Literature 47(2), 448–474.

[rego12440-bib-0023] Davison AC , Hinkley DV (1997) Bootstrap Methods and Their Application. Cambridge University Press, Los Angeles.

[rego12440-bib-0024] Declerck C , Boone C , Emonds G (2013) When Do People Cooperate? The Neuroeconomics of Prosocial Decision Making. Brain and Cognition 81(1), 95–117. 23174433 10.1016/j.bandc.2012.09.009

[rego12440-bib-0025] Desclaux A , Badji D , Ndione AG , Sow K (2017) Accepted Monitoring or Endured Quarantine? Ebola Contacts' Perceptions in Senegal. Social Science & Medicine 178, 38–45. 28192745 10.1016/j.socscimed.2017.02.009

[rego12440-bib-0026] Devine D , Gaskell J , Jennings W , Stoker G (2020) Covid19? Trust and the Coronavirus Pandemic: What Are the Consequences of and for Trust? An Early Review of the Literature. Political Studies Review 19, 274–285. 10.1177/1478929920948684 PMC7424609 35082554

[rego12440-bib-0027] Dryhurst S , Schneider CR , Kerr J *et al*. (2020) Risk Perceptions of COVID‐19 Around the World. Journal of Risk Research 23(7–8), 994–1006.

[rego12440-bib-0028] Etienne J (2013) Ambiguity and Relational Signals in Regulator–Regulatee Relationships. Regulation & Governance 7(1), 30–47.

[rego12440-bib-0029] Feld LP , Frey BS (2002) Trust Breeds Trust: How Taxpayers Are Treated. Economics of Governance 3(2), 87–99.

[rego12440-bib-0030] Flaxman S , Mishra S , Axel G *et al*. (2020) Estimating the Effects of Non‐pharmaceutical Interventions on COVID‐19 in Europe. Nature 584(7820), 257–261. 32512579 10.1038/s41586-020-2405-7

[rego12440-bib-0076] Grimmelikhuijsen S , Knies E (2017) Validating a scale for citizen trust in government organizations. International Review of Administrative Sciences 83(3), 583–601. 10.1177/0020852315585950

[rego12440-bib-0031] Gunningham N , Sinclair D (2009) Regulation and the Role of Trust: Reflections from the Mining Industry. Journal of Law and Society 36(2), 167–194.

[rego12440-bib-0032] Harper CA , Satchell LP , Fido D , Latzman RD (2020) Functional Fear Predicts Public Health Compliance in the COVID‐19 Pandemic. International Journal of Mental Health and Addiction, 1–14. 10.1007/s11469-020-00281-5 PMC7185265 32346359

[rego12440-bib-0033] Jakobsen M , Jensen R (2015) Common Method Bias in Public Management Studies. International Public Management Journal 18(1), 3–30.

[rego12440-bib-0034] Jørgensen FJ , Bor A , Petersen MB (2020) Compliance Without Fear: Individual‐level Predictors of Protective Behavior during the First Wave of the COVID‐19 Pandemic. Working Paper. PsyArXiv. 10.31234/osf.io/uzwgf. PMC8250211 33763971

[rego12440-bib-0036] Korsgaard MA (2018) Reciprocal Trust: A Self‐reinforcing Dynamic Process. In: Rosalind SH , Ann‐Marie NI , Sitkin SB (eds) The Routledge Companion to Trust, pp. 14–22. Routledge, London.

[rego12440-bib-0037] Levi M , Stoker L (2000) Political Trust and Trustworthiness. Annual Review of Political Science 3(1), 475–507.

[rego12440-bib-0038] Lim S , Nakazato H (2020) The Emergence of Risk Communication Networks and the Development of Citizen Health‐related Behaviors during the COVID‐19 Pandemic: Social Selection and Contagion Processes. International Journal of Environmental Research and Public Health 17(11), 4148. 10.3390/ijerph17114148 PMC7312553 32532029

[rego12440-bib-0039] Lindenberg S (2001) Intrinsic Motivation in a New Light. Kyklos 54(2–3), 317–342.

[rego12440-bib-0040] Lindenberg S (2006) Prosocial Behavior, Solidarity, and Framing Processes. Solidarity and Prosocial Behavior, pp. 23–44. Springer, Boston.

[rego12440-bib-0041] Lindenberg S (2017) The Dependence of Human Cognitive and Motivational Processes on Institutional Systems. Social Dilemmas, Institutions and the Evolution of Cooperation, pp. 85–106. De Gruyter Oldenbourg, Berlin.

[rego12440-bib-0042] Lindenberg S , Six F , Keizer K (2021) Social Contagion and Goal Framing: The Sustainability of Rule Compliance. In: Van Rooij B , Sokol DD (eds) Cambridge Handbook of Compliance. Cambridge University Press, Cambridge.

[rego12440-bib-0043] Lindenberg S , Steg L (2007) Normative, Gain and Hedonic Goal Frames Guiding Environmental Behavior. Journal of Social Issues 63(1), 117–137.

[rego12440-bib-0044] Matsumoto Y , Yamagishi T , Yang L , Kiyonari T (2016) Prosocial Behavior Increases with Age across Five Economic Games. PLoS One 11(7), e0158671. 27414803 10.1371/journal.pone.0158671 PMC4945042

[rego12440-bib-0045] McKay L (2021) Levelled‐up or Forgotten People? Place and Public Perceptions of Bias in the Johnson Government. *TrustGov* . [Last accessed 6 May 2021.] Available from URL: https://trustgov.net/trustgov-blog.

[rego12440-bib-0046] Meier KJ , O'Toole LJ (2012) Subjective Organizational Performance and Measurement Error: Common Source Bias and Spurious Relationships. Journal of Public Administration Research and Theory 23(2), 429–456.

[rego12440-bib-0047] Murphy K (2004) The Role of Trust in Nurturing Compliance: A Study of Accused Tax Avoiders. Law and Human Behavior 28(2), 187–209. 15141778 10.1023/b:lahu.0000022322.94776.ca

[rego12440-bib-0048] Murphy K , Tyler TR , Curtis A (2009) Nurturing Regulatory Compliance: Is Procedural Justice Effective When People Question the Legitimacy of the Law? Regulation & Governance 3(1), 1–26.

[rego12440-bib-0049] Nivette A , Ribeaud D , Murray A *et al*. (2021) Non‐compliance with COVID‐19‐Related Public Health Measures among Young Adults in Switzerland: Insights from a Longitudinal Cohort Study. Social Science & Medicine 268, 268. 10.1016/j.socscimed.2020.113370 PMC7493799 32980677

[rego12440-bib-0050] OECD (2014) OECD Best Practice Principles for Regulatory Enforcement and Inspections. OECD Publishing, Paris.

[rego12440-bib-0051] OECD (2017) OECD Guidelines on Measuring Trust. OECD Publishing, Paris.

[rego12440-bib-0052] Oosterhoff B , Palmer C (2020) Psychological Correlates of News Monitoring, Social Distancing, Disinfecting, and Hoarding Behaviors among US Adolescents during the COVID‐19 Pandemic. Preprint. PsyArXiv. 10.31234/osf.io/rpcy4. PMC7325067 32597925

[rego12440-bib-0053] Pfattheicher S , Nockur L , Böhm R , Sassenrath C , Petersen MB (2020) The Emotional Path to Action: Empathy Promotes Physical Distancing and Wearing Face Masks during the COVID‐19 Pandemic. Preprint. PsyArXiv. 10.31234/osf.io/y2cg5. 32993455

[rego12440-bib-0054] Podsakoff PM , MacKenzie SB , Podsakoff NP (2012) Sources of Method Bias in Social Science Research and Recommendations on How to Control It. Annual Review of Psychology 63, 539–569. 10.1146/annurev-psych-120710-100452 21838546

[rego12440-bib-0055] Proszowska DK (2020) Multilevel Trust and the “Intelligent Lockdown” in the Netherlands. Paper presented at the GOVTRUST Annual Symposium 2021, 29 January 2021.

[rego12440-bib-0056] Proszowska DK , Jansen G , de Vries PW (2020) COVID‐19 en de “intelligente lockdown” in de ogen van de burgers: deel 3: Meningen over de corona‐app.

[rego12440-bib-0057] Raude J , Debin M , Souty C , Guerrisi C , Turbelin C , Falchi A , et al. 2020. Are People Excessively Pessimistic about the Risk of Coronavirus Infection? Preprint. PsyArXiv. 10.31234/osf.io/364qj.

[rego12440-bib-0058] Rihoux B , Ragin CC (2008) Configurational Comparative Methods: Qualitative Comparative Analysis (QCA) and Related Techniques. Sage Publications, London.

[rego12440-bib-0059] Robertson E , Hershenfield K , Grace SL , Stewart DE (2004) The Psychosocial Effects of Being Quarantined Following Exposure to SARS: A Qualitative Study of Toronto Health Care Workers. The Canadian Journal of Psychiatry 49(6), 403–407. 15283537 10.1177/070674370404900612

[rego12440-bib-0060] Rubin GJ , Amlot R , Page L , Wessely S (2009) Public Perceptions, Anxiety, and Behaviour Change in Relation to the Swine Flu Outbreak: Cross Sectional Telephone Survey. BMJ 339, b2651–b2651. 10.1136/bmj.b2651. 19574308 PMC2714687

[rego12440-bib-0061] Schneider CR , Freeman ALJ , Spiegelhalter D , van der Linden S (2021) The Effects of Quality of Evidence Communication on Perception of Public Health Information about COVID‐19: Two Randomised Controlled Trials. *medRxiv* . 10.1371/journal.pone.0259048 PMC8598038 34788299

[rego12440-bib-0062] Sciensano (2020) COVID‐19 – Epidemiologisch Bulletin van 14 July 2020. Available from URL: https://covid-19.sciensano.be/sites/default/files/Covid19/COVID-19_Daily%20report_20200714%20-%20NL.pdf [last accessed September 16 2020].

[rego12440-bib-0063] Seddon JJM , Currie WL (2013) Cloud Computing and Trans‐border Health Data: Unpacking U.S. and EU Healthcare Regulation and Compliance. Health Policy and Technology 2(4), 229–241.

[rego12440-bib-0064] Six F , Verhoest K (2017) Trust in Regulatory Regimes: Scoping the Field. Trust in Regulatory Regimes. Edward Elgar Publishing, Cheltenham.

[rego12440-bib-0065] Tang C S‐k , Wong C‐y (2003) An Outbreak of the Severe Acute Respiratory Syndrome: Predictors of Health Behaviors and Effect of Community Prevention Measures in Hong Kong, China. American Journal of Public Health 93(11), 1887–1888. 14600058 10.2105/ajph.93.11.1887 PMC1448068

[rego12440-bib-0066] Tenbrunsel AE , Messick DM (1999) Sanctioning Systems, Decision Frames, and Cooperation. Administrative Science Quarterly 44(4), 684–707.

[rego12440-bib-0067] Trinkner R , Tyler TR (2016) Legal Socialization: Coercion Versus Consent in an Era of Mistrust. Annual Review of Law and Social Science 12, 417–439.

[rego12440-bib-0068] Tyler TR (2006) Why People Obey the Law?. Princeton University Press, Princeton, NJ.

[rego12440-bib-0069] van Rooij B , de Bruijn AL , Reinders Folmer C , Kooistra EB , Kuiper ME , Brownlee M , et al. (2020) Compliance with COVID‐19 Mitigation Measures in the United States (April 22, 2020). Amsterdam Law School Research Paper No. 2020‐21, General Subserie Research Paper No. 2020‐03, UC Irvine School of Law Research Paper No. 2020‐33. Available at SSRN: https://ssrn.com/abstract=3582626 or 10.2139/ssrn.3582626

[rego12440-bib-0070] Velez MA , Lopez MC (2013) Rules Compliance and Age: Experimental Evidence with Fishers from the Amazon River. Ecology and Society 18(3), 10–33.

[rego12440-bib-0071] Vinck P , Pham PN , Bindu KK , Bedford J , Nilles EJ (2019) Institutional Trust and Misinformation in the Response to the 2018–19 Ebola Outbreak in North Kivu, DR Congo: A Population‐based Survey. The Lancet Infectious Diseases 19(5), 529–536. 30928435 10.1016/S1473-3099(19)30063-5

[rego12440-bib-0075] Weber M (1978) Economy and Society: An Outline of Interpretive Sociology. University of California Press, Berkeley.

[rego12440-bib-0072] Webster RK , Brooks SK , Smith LE , Woodland L , Wessely S , Rubin GJ (2020) How to Improve Adherence with Quarantine: Rapid Review of the Evidence. Public Health 182, 163–169. 32334182 10.1016/j.puhe.2020.03.007 PMC7194967

[rego12440-bib-0073] Wise T , Zbozinek TD , Michelini G , Hagan CC (2020) Changes in Risk Perception and Protective Behavior during the First Week of the COVID‐19 Pandemic in the United States. PsyArXiv. 10.31234/osf.io/dz428. PMC7540790 33047037

[rego12440-bib-0074] Zettler I , Schild C , Lilleholt L , Kroencke L , Utesch T , Moshagen M , et al. (2020) The Role of Personality in COVID‐19 Related Perceptions, Evaluations, and Behaviors: Findings across Five Samples, Nine Traits, and 17 Criteria. Preprint. PsyArXiv.

