# Supplementary material for: What drives compliance with COVID‐19 measures over time? Explaining changing impacts with Goal Framing Theory
Source: Regul Gov. 2021 Oct 7:10.1111/rego.12440. Online ahead of print. doi: 10.1111/rego.12440 (PMC8661714; doi:10.1111/rego.12440)
Supplement: Supplementary file 1 — Appendix S1. Supporting Information. [file REGO-9999-0-s001.docx]

Online supplemental information

Appendix

What drives compliance with COVID-19 measures over time? 
Studying the changing impact of factors with Goal Framing Theory

Table of Contents

[Appendix 1 - Descriptives 2](#_Toc80263980)

[Appendix 2 - Robustness checks 3](#_Toc80263981)

[New dependent variable 3](#_Toc80263982)

[Ordered logit analyses 5](#_Toc80263983)

[Appendix 3 - Individual Steps Regression 6](#_Toc80263984)

[Wave 1 6](#_Toc80263985)

[Wave 2 7](#_Toc80263986)

[Wave 3 8](#_Toc80263987)

[Appendix 4 - Models without Rule Effectiveness 9](#_Toc80263988)

[Final model 9](#_Toc80263989)

[Wave 1 10](#_Toc80263990)

[Wave 2 11](#_Toc80263991)

[Wave 3 12](#_Toc80263992)

[Robustness check 13](#_Toc80263993)

[New dependent variable 13](#_Toc80263994)

[Ordered logit analyses 14](#_Toc80263995)

# Appendix 1 - Descriptives

| **Variables** | **Population** | **Survey Wave 1** | **Survey Wave 2** | **Survey Wave 3** |
| --- | --- | --- | --- | --- |
| *Gender* |  |  |  |  |
| Male | 49.1 | 42.1 | 40.6 | 40.4 |
| Female | 50.9 | 57.9 | 59.4 | 59.6 |
| *Age* |  |  |  |  |
| 15-24 | 13.0 | 13.1 | 11.5 | 8.1 |
| 25-34 | 14.7 | 17.7 | 16.6 | 13.9 |
| 35-44 | 15.2 | 18.4 | 18.1 | 15.8 |
| 45-54 | 16.7 | 18.6 | 18.5 | 18.2 |
| 55-64 | 16.3 | 17.1 | 17.9 | 20.5 |
| 65+ | 24.1 | 15.1 | 17.4 | 23.5 |
| *Province* |  |  |  |  |
| Antwerp | 28.0 | 32.4 | 33.6 | 34.8 |
| Flemish Brabant | 17.2 | 16.9 | 16.9 | 17.5 |
| West Flanders | 18.4 | 15.8 | 14.9 | 14.4 |
| East Flanders | 23.0 | 22.4 | 22.2 | 21.4 |
| Limburg | 13.4 | 12.5 | 12.4 | 11.9 |
| *Education* |  |  |  |  |
| Primary education | 11.3 | 4.0 | 3.4 | 3.9 |
| Secondary education | 55.8 | 51.9 | 50.7 | 49.8 |
| Bachelor-level education | 16.2 | 22.1 | 22.8 | 22.9 |
| Masters/PhD-level education | 16.7 | 21.9 | 23.1 | 23.3 |

*Weighted descriptive table (in %) of sociodemographic variables*

# Appendix 2 - Robustness checks

## New dependent variable

**This robustness test utilizes more specific compliance questions from Survey Wave 2. The Dependent Variable is based on several variables available only in this wave: 1) Change of behaviour in public spaces/shops, 2) last kiss/hand, 3) social distancing.**

For this new variable we use 3 questions (everything between ## was omitted for people living alone):

Q38_3: On a scale from 0 to 10, please indicate to what extent you have adapted your behaviour to the new guidelines. - In public places/in shops

Q10BenQ63B: When was the last time you shook hands or kissed #anyone other than a roommate# after April 21?

Q314andQ315: Do you keep one and a half metres away from anyone #other than your housemates# (unless the situation makes this impossible for reasons beyond your control)?

Based on these questions we designed 3 categories of compliance (low-medium-high), which result in a reasonable distribution. Low compliance: ((Q38_3 < 10)); Medium compliance: ((Q38_3 >= 10) AND ((Q10BenQ63B>1) OR (Q314andQ315 < 5))); High compliance: ((Q38_3 >= 10) AND (Q10BenQ63B=1) AND (Q314enQ315 = 5))

Overall, this robustness check yields **the same significant effects as the basic model for survey wave 2** (see middle column in table 3 in the paper) for the variables rule appropriateness, observed respect for rules, risk severity. Like in the basic model, trust in government is not significant. The very small effects at significance level 0,05 of risk proximity and rule effectiveness in the basic model (see table 3 in the paper) turn out to be no longer significant in the robustness check.

|  | Wave 2 |
| --- | --- |
| Age | 1.036***  (0.003) |
| Female | 1.425***  (0.062) |
| Alone | 0.673**  (0.084) |
| Education | 0.976  (0.037) |
| Pro-Socialness | 1.100  (0.147) |
| Trust in Government | 0.745  (0.140) |
| Rule Effectiveness | 1.510  (0.160) |
| Rule Appropriateness | 2.306***  (0.103) |
| Observed respect for rules | 19.381***  (0.175) |
| Fear: risk severity | 3.192***  (0.167) |
| Fear:  risk proximity | 1.337  (0.120) |
| Pleasure & Happiness | 1.120  (0.046) |
| Worsened income position | 1.141  (0.072) |
|  |  |
| Nagelkerke | 0.163 |
| logLik | -3924.137 |
| McFadden | 0.094 |

*Weighted results ordered logit model with bootstrapping analysis without replacement*
*Sample sizes = 10,000, number of resampling = 10,000. Shown results are average odds ratios over all resamples and standard errors in parentheses.* 
*Level of significance: ***p < 0.001, **p <0.010, *p<0.050, p<0.100.*

## Ordered logit analyses

In this robustness check the dependent variable is the same as in the model in the main text but we analysed it by means of an ordered logit model. There are small differences, with ‘trust in government’ becoming negatively significant in Wave 2 (whereas in the OLS model it only becomes significant in Wave 3), and some socio-demographic variables also show a negative significant effect (the variable ‘alone in Wave 1 and Wave 2; and the variable education in Wave 3).

|  | Wave 1 | Wave 2 | Wave 3 |
| --- | --- | --- | --- |
| Age | 1.023***  (0.002) | 1.023***  (0.002) | 1.033***  (0.002) |
| Female | 1.370***  (0.049) | 1.467***  (0.050) | 1.850***  (0.071) |
| Alone | 0.780*  (0.073) | 0.788*  (0.071) | 1.003  (0.088) |
| Education | 0.991  (0.029) | 0.969  (0.030) | 0.854*  (0.042) |
| Pro-Socialness | 1.235  (0.118) | 1.095  (0.121) | 1.838**  (0.160) |
| Trust in Government | 0.847  (0.115) | 0.653*  (0.113) | 0.565*  (0.172) |
| Rule Effectiveness | 1.293  (0.135) | 1.544*  (0.133) | 0.777  (0.192) |
| Rule Appropriateness | 2.523***  (0.086) | 2.361***  (0.084) | 4.088***  (0.122) |
| Observed respect for rules | 56.293***  (0.160) | 34.902***  (0.156) | 13.725***  (0.183) |
| Fear: risk severity | 3.536***  (0.157) | 3.961***  (0.146) | 4.024***  (0.211) |
| Fear:  risk proximity | 1.278  (0.100) | 1.371*  (0.097) | 2.347***  (0.137) |
| Pleasure & Happiness | 1.148**^.^**  (0.038) | 1.131**^.^**  (0.038) | 1.124  (0.063) |
| Worsened income position | 1.159**^.^**  (0.055) | 1.184**^.^**  (0.057) | 1.312  (0.159) |
|  |  |  |  |
| Nagelkerke | 0.185 | 0.177 | 0.245 |
| logLik | -7885.919 | -7610.132 | -4863,670 |
| McFadden | 0.072 | 0.068 | 0.077 |

*Weighted results ordered logit model with bootstrapping analysis without replacement*
*Sample sizes = 10,000, number of resampling = 10,000. Shown results are average odds ratios over all resamples and standard errors in parentheses.* 
*Level of significance: ***p < 0.001, **p <0.010, *p<0.050, p<0.100*

# Appendix 3 - Individual Steps Regression

## Wave 1

|  | 0 | 1 | 2 | 3 | 4 | 5 | 6 | 7 | 8 | 9 |
| --- | --- | --- | --- | --- | --- | --- | --- | --- | --- | --- |
| Age | 0.165*** | 0.161*** | 0.151*** | 0.155*** | 0.152*** | 0.125*** | 0.110*** | 0.112*** | 0.112*** | 0.115*** |
| Female | 0.100*** | 0.098*** | 0.097*** | 0.097*** | 0.086*** | 0.069** | 0.063** | 0.061** | 0.060** | 0.066** |
| Alone | -0.066*** | -0.064*** | -0.062*** | -0.066** | -0.063* | -0.043**^.^** | -0.042**^.^** | -0.041**^.^** | -0.044**^.^** | -0.029 |
| Education | 0.040* | 0.038* | 0.029**^.^** | 0.028 | 0.021 | 0.003 | 0.005 | 0.006 | 0.008 | 0.007 |
| Pro-Socialness |  | 0.076*** | 0.064** | 0.057* | 0.057* | 0.040**^.^** | 0.037 | 0.034 | 0.033 | 0.033 |
| Trust in Government (to handle COVID 19) |  |  | 0.059** | 0.013 | 0.005 | -0.017 | -0.015 | -0.013 | -0.010 | -0.005 |
| Rule Effectiveness |  |  |  | 0.076* | 0.074* | 0.038 | 0.021 | 0.019 | 0.022 | 0.024 |
| Rule Appropriateness |  |  |  |  | 0.120*** | 0.125*** | 0.109*** | 0.110*** | 0.113*** | 0.120*** |
| Observed respect for rules |  |  |  |  |  | 0.330*** | 0.326*** | 0.329*** | 0.330*** | 0.327*** |
| Fear: risk severity |  |  |  |  |  |  | 0.106*** | 0.099*** | 0.099*** | 0.108*** |
| Fear: risk proximity |  |  |  |  |  |  |  | 0.038 | 0.036 | 0.030 |
| Pleasure & Happiness |  |  |  |  |  |  |  |  | 0.042**^.^** | 0.040 |
| Worsened income position |  |  |  |  |  |  |  |  |  | 0.018 |
|  |  |  |  |  |  |  |  |  |  |  |
| R^2^ | 0.036 | 0.042 | 0.045 | 0.051 | 0.065 | 0.164 | 0.172 | 0.174 | 0.176 | 0.187 |
| Adjusted R^2^ | 0.036 | 0.042 | 0.045 | 0.050 | 0.064 | 0.162 | 0.171 | 0.172 | 0.174 | 0.184 |

*Weighted regression result (standardized coefficients) in bootstrapping analysis without replacement*
*Sample sizes = 10,000, number of resampling = 10,000. Shown results are averages over all resamples.* 
*Level of significance: ***p < 0.001, **p <0.010, *p<0.050, p<0.100.*

|  | 0 | 1 | 2 | 3 | 4 | 5 | 6 | 7 | 8 | 9 |
| --- | --- | --- | --- | --- | --- | --- | --- | --- | --- | --- |
| Age | 0.206*** | 0.201*** | 0.195*** | 0.191*** | 0.186*** | 0.165*** | 0.137*** | 0.135*** | 0.137*** | 0.120*** |
| Female | 0.118*** | 0.116*** | 0.115*** | 0.116*** | 0.109*** | 0.092*** | 0.083*** | 0.082*** | 0.080*** | 0.084*** |
| Alone | -0.073*** | -0.074*** | -0.074*** | -0.073*** | -0.069*** | -0.054** | -0.051** | -0.050** | -0.054** | -0.035**^.^** |
| Education | 0.019 | 0.015 | 0.009 | 0.003 | -0.001 | -0.015 | -0.011 | -0.008 | -0.008 | -0.001 |
| Pro-Socialness |  | 0.054** | 0.045* | 0.040* | 0.041* | 0.025 | 0.024 | 0.024 | 0.023 | 0.024 |
| Trust in Government (to handle COVID 19) |  |  | 0.048** | -0.024 | -0.037**^.^** | -0.054** | -0.050* | -0.051* | -0.048* | -0.043**^.^** |
| Rule Effectiveness |  |  |  | 0.118*** | 0.102*** | 0.073** | 0.051* | 0.050* | 0.052* | 0.054* |
| Rule Appropriateness |  |  |  |  | 0.116*** | 0.136*** | 0.118*** | 0.115*** | 0.121*** | 0.125*** |
| Observed respect for rules |  |  |  |  |  | 0.292*** | 0.294*** | 0.298*** | 0.299*** | 0.309*** |
| Fear: risk severity |  |  |  |  |  |  | 0.139*** | 0.130*** | 0.129*** | 0.136*** |
| Fear: risk proximity |  |  |  |  |  |  |  | 0.048** | 0.047** | 0.044* |
| Pleasure & Happiness |  |  |  |  |  |  |  |  | 0.039* | 0.034**^.^** |
| Worsened income position |  |  |  |  |  |  |  |  |  | 0.021 |
|  |  |  |  |  |  |  |  |  |  |  |
| R^2^ | 0.051 | 0.054 | 0.056 | 0.065 | 0.076 | 0.154 | 0.173 | 0.175 | 0.177 | 0.183 |
| Adjusted R^2^ | 0.051 | 0.054 | 0.056 | 0.064 | 0.075 | 0.153 | 0.173 | 0.174 | 0.175 | 0.181 |

## Wave 2

*Weighted regression result (standardized coefficients) in bootstrapping analysis without replacement*
*Sample sizes = 10,000, number of resampling = 10,000. Shown results are averages over all resamples.* 
*Level of significance: ***p < 0.001, **p <0.010, *p<0.050, p<0.100.*

## Wave 3

|  | 0 | 1 | 2 | 3 | 4 | 5 | 6 | 7 | 8 | 9 |
| --- | --- | --- | --- | --- | --- | --- | --- | --- | --- | --- |
| Age | 0.282*** | 0.280*** | 0.276*** | 0.276*** | 0.253*** | 0.224*** | 0.190*** | 0.197*** | 0.197*** | 0.200*** |
| Female | 0.170*** | 0.169*** | 0.174*** | 0.174*** | 0.163*** | 0.134*** | 0.125*** | 0.120*** | 0.119*** | 0.119*** |
| Alone | 0.009 | 0.008 | 0.009 | 0.009 | 0.013 | 0.024 | 0.024 | 0.020 | 0.020 | 0.022 |
| Education | -0.016 | -0.031* | -0.033* | -0.036* | -0.047** | -0.026 | -0.031 | -0.024 | -0.024 | -0.024 |
| Pro-Socialness |  | 0.130*** | 0.128*** | 0.127*** | 0.119*** | 0.102*** | 0.099*** | 0.096*** | 0.096*** | 0.093*** |
| Trust in Government (to handle COVID 19) |  |  | 0.009 | -0.049* | -0.051* | -0.090** | -0.086** | -0.083** | -0.083** | -0.077* |
| Rule Effectiveness |  |  |  | 0.083*** | 0.080*** | 0.037 | -0.004 | -0.010 | -0.011 | -0.008 |
| Rule Appropriateness |  |  |  |  | 0.217*** | 0.242*** | 0.210*** | 0.206*** | 0.206*** | 0.203*** |
| Observed respect for rules |  |  |  |  |  | 0.266*** | 0.267*** | 0.275*** | 0.275*** | 0.282*** |
| Fear: risk severity |  |  |  |  |  |  | 0.175*** | 0.143*** | 0.143*** | 0.145*** |
| Fear: risk proximity |  |  |  |  |  |  |  | 0.108*** | 0.108*** | 0.105*** |
| Pleasure & Happiness |  |  |  |  |  |  |  |  | 0.015 | 0.016 |
| Worsened income position |  |  |  |  |  |  |  |  |  | 0.011 |
|  |  |  |  |  |  |  |  |  |  |  |
| R^2^ | 0.095 | 0.110 | 0.111 | 0.115 | 0.157 | 0.199 | 0.223 | 0.230 | 0.230 | 0.233 |
| Adjusted R^2^ | 0.095 | 0.110 | 0.110 | 0.114 | 0.157 | 0.197 | 0.221 | 0.227 | 0.228 | 0.230 |

*Weighted regression result (standardized coefficients) in bootstrapping analysis without replacement*
*Sample sizes = 10,000, number of resampling = 10,000. Shown results are averages over all resamples.* 
*Level of significance: ***p < 0.001, **p <0.010, *p<0.050, p<0.100.*

# Appendix 4 - Models without Rule Effectiveness

## Final model

|  | Wave 1 | Wave 2 | Wave 3 |
| --- | --- | --- | --- |
| Age | 0.115*** | 0.120*** | 0.200*** |
| Female | 0.066** | 0.083*** | 0.120*** |
| Alone | -0.028 | -0.035**^.^** | 0.021 |
| Education | 0.008 | 0.002 | -0.024 |
| Pro-Socialness | 0.034 | 0.027 | 0.091*** |
| Trust in Government (to handle the COVID 19 crisis) | 0.006 | -0.014 | -0.083*** |
| Rule  Appropriateness | 0.119*** | 0.131*** | 0.204*** |
| Observed respect for rules | 0.328*** | 0.314*** | 0.280*** |
| Fear: risk severity | 0.110*** | 0.142*** | 0.145*** |
| Fear:  risk proximity | 0.031 | 0.045* | 0.105*** |
| Pleasure & Happiness | 0.038 | 0.033 | 0.016 |
| Worsened Income position | 0.018 | 0.021 | 0.013 |
|  |  |  |  |
| R^2^ | 0.185 | 0.181 | 0.233 |
| Adjusted R^2^ | 0.182 | 0.179 | 0.230 |

*Weighted regression result (standardized coefficients) in bootstrapping analysis without replacement*
*Sample sizes = 10.000. number of resampling = 10.000. Shown results are averages over all resamples.* 
*Level of significance: ***p < 0.001, **p <0.010, *p<0.050, p<0.100.*

## Wave 1

|  | 0 | 1 | 2 | 3 | 4 | 5 | 6 | 7 | 8 |
| --- | --- | --- | --- | --- | --- | --- | --- | --- | --- |
| Age | 0.165*** | 0.161*** | 0.151*** | 0.153*** | 0.126*** | 0.111*** | 0.112*** | 0.113*** | 0.115*** |
| Female | 0.100*** | 0.098*** | 0.097*** | 0.089*** | 0.070** | 0.064** | 0.062** | 0.061** | 0.066** |
| Alone | -0.066*** | -0.064*** | -0.062*** | -0.063* | -0.043**^.^** | -0.042**^.^** | -0.040**^.^** | -0.044**^.^** | -0.028 |
| Education | 0.040* | 0.038* | 0.029**^.^** | 0.024 | 0.005 | 0.006 | 0.007 | 0.008 | 0.008 |
| Pro-Socialness |  | 0.076*** | 0.064** | 0.061* | 0.044**^.^** | 0.039 | 0.036 | 0.035 | 0.034 |
| Trust in Government (to handle COVID 19) |  |  | 0.059** | 0.043**^.^** | 0.002 | -0.005 | -0.003 | 0.001 | 0.006 |
| Rule Appropriateness |  |  |  | 0.120*** | 0.124*** | 0.109*** | 0.109*** | 0.113*** | 0.119*** |
| Observed respect for rules |  |  |  |  | 0.333*** | 0.327*** | 0.329*** | 0.331*** | 0.328*** |
| Fear: risk severity |  |  |  |  |  | 0.109*** | 0.102*** | 0.102*** | 0.110*** |
| Fear: risk proximity |  |  |  |  |  |  | 0.039 | 0.036 | 0.031 |
| Pleasure & Happiness |  |  |  |  |  |  |  | 0.040**^.^** | 0.038 |
| Worsened income position |  |  |  |  |  |  |  |  | 0.018 |
|  |  |  |  |  |  |  |  |  |  |
| R^2^ | 0.036 | 0.042 | 0.045 | 0.061 | 0.162 | 0.171 | 0.173 | 0.175 | 0.185 |
| Adjusted R^2^ | 0.036 | 0.042 | 0.045 | 0.059 | 0.161 | 0.169 | 0.171 | 0.173 | 0.182 |

*Weighted regression result (standardized coefficients) in bootstrapping analysis without replacement*
*Sample sizes = 10.000. number of resampling = 10.000. Shown results are averages over all resamples.* 
*Level of significance: ***p < 0.001, **p <0.010, *p<0.050, p<0.100.*

## Wave 2

|  | 0 | 1 | 2 | 3 | 4 | 5 | 6 | 7 | 8 |
| --- | --- | --- | --- | --- | --- | --- | --- | --- | --- |
| Age | 0.206*** | 0.201*** | 0.195*** | 0.188*** | 0.166*** | 0.137*** | 0.135*** | 0.137*** | 0.120*** |
| Female | 0.118*** | 0.116*** | 0.115*** | 0.109*** | 0.092*** | 0.083*** | 0.081*** | 0.080*** | 0.083*** |
| Alone | -0.073*** | -0.074*** | -0.074*** | -0.069*** | -0.054** | -0.051** | -0.051** | -0.054** | -0.035**^.^** |
| Education | 0.019 | 0.015 | 0.009 | 0.002 | -0.013 | -0.009 | -0.006 | -0.006 | 0.002 |
| Pro-Socialness |  | 0.054** | 0.045* | 0.045* | 0.028**^.^** | 0.025 | 0.025 | 0.025 | 0.027 |
| Trust in Government (to handle COVID 19) |  |  | 0.048** | 0.024 | -0.012 | -0.022 | -0.024 | -0.019 | -0.014 |
| Rule Appropriateness |  |  |  | 0.123*** | 0.142*** | 0.122*** | 0.119*** | 0.125*** | 0.131*** |
| Observed respect for rules |  |  |  |  | 0.297*** | 0.297*** | 0.301*** | 0.303*** | 0.314*** |
| Fear: risk severity |  |  |  |  |  | 0.144*** | 0.134*** | 0.135*** | 0.142*** |
| Fear: risk proximity |  |  |  |  |  |  | 0.049** | 0.048** | 0.045* |
| Pleasure & Happiness |  |  |  |  |  |  |  | 0.039* | 0.033 |
| Worsened income position |  |  |  |  |  |  |  |  | 0.021 |
|  |  |  |  |  |  |  |  |  |  |
| R^2^ | 0.051 | 0.054 | 0.056 | 0.069 | 0.150 | 0.172 | 0.174 | 0.176 | 0.181 |
| Adjusted R^2^ | 0.051 | 0.054 | 0.056 | 0.069 | 0.150 | 0.171 | 0.173 | 0.175 | 0.179 |

*Weighted regression result (standardized coefficients) in bootstrapping analysis without replacement*
*Sample sizes = 10.000. number of resampling = 10.000. Shown results are averages over all resamples.* 
*Level of significance: ***p < 0.001, **p <0.01,. *p<0.050, p<0.100.*

## Wave 3

|  | 0 | 1 | 2 | 3 | 4 | 5 | 6 | 7 | 8 |
| --- | --- | --- | --- | --- | --- | --- | --- | --- | --- |
| Age | 0.282*** | 0.280*** | 0.276*** | 0.253*** | 0.224*** | 0.189*** | 0.196*** | 0.196*** | 0.200*** |
| Female | 0.170*** | 0.169*** | 0.174*** | 0.163*** | 0.135*** | 0.126*** | 0.120*** | 0.120*** | 0.120*** |
| Alone | 0.009 | 0.008 | 0.009 | 0.014 | 0.023 | 0.023 | 0.019 | 0.019 | 0.021 |
| Education | -0.016 | -0.031* | -0.033* | -0.045** | -0.026 | -0.033 | -0.026 | -0.026 | -0.024 |
| Pro-Socialness |  | 0.130*** | 0.128*** | 0.120*** | 0.102*** | 0.098*** | 0.094*** | 0.095*** | 0.091*** |
| Trust in Government (to handle COVID 19) |  |  | 0.009 | 0.005 | -0.063** | -0.089*** | -0.090*** | -0.090*** | -0.083*** |
| Rule Appropriateness |  |  |  | 0.219*** | 0.244*** | 0.211*** | 0.208*** | 0.208*** | 0.204*** |
| Observed respect for rules |  |  |  |  | 0.266*** | 0.265*** | 0.273*** | 0.274*** | 0.280*** |
| Fear: risk severity |  |  |  |  |  | 0.175*** | 0.143*** | 0.143*** | 0.145*** |
| Fear: risk proximity |  |  |  |  |  |  | 0.108*** | 0.108*** | 0.105*** |
| Pleasure & Happiness |  |  |  |  |  |  |  | 0.016 | 0.016 |
| Worsened income position |  |  |  |  |  |  |  |  | 0.013 |
|  |  |  |  |  |  |  |  |  |  |
| R^2^ | 0.095 | 0.110 | 0.111 | 0.154 | 0.198 | 0.223 | 0.230 | 0.230 | 0.233 |
| Adjusted R^2^ | 0.095 | 0.110 | 0.110 | 0.154 | 0.196 | 0.221 | 0.228 | 0.228 | 0.230 |

*Weighted regression result (standardized coefficients) in bootstrapping analysis without replacement*
*Sample sizes = 10.000. number of resampling = 10.000. Shown results are averages over all resamples.* 
*Level of significance: ***p < 0.001, **p <0.010, *p<0.050, p<0.100.*

## Robustness check

### New dependent variable

**Robustness test utilizes more specific compliance questions from Survey Wave 2. The Dependent Variable is based on several variables available only in this wave: 1) Change of behaviour in public spaces/shops, 2) last kiss/hand, 3) social-distancing.**

For this new variable we use 3 questions (everything between ## was omitted for people living alone):

Q38_3: On a scale from 0 to 10, please indicate to what extent you have adapted your behaviour to the new guidelines. - In public places/in shops

Q10BenQ63B: When was the last time you shook hands or kissed #anyone other than a roommate# after April 21?

Q314andQ315: Do you keep one and a half metres away from anyone #other than your housemates# (unless the situation makes this impossible for reasons beyond your control)?

**Based on these questions we designed 3 categories of compliance (low-medium-high), which result in a reasonable distribution.** Low compliance: ((Q38_3 < 10)); Medium compliance: ((Q38_3 >= 10) AND ((Q10BenQ63B>1) OR (Q314andQ315 < 5))); High compliance: ((Q38_3 >= 10) AND (Q10BenQ63B=1) AND (Q314enQ315 = 5))

|  | Wave 2 |
| --- | --- |
| Age | 1.036*** |
| Female | 1.421*** |
| Alone | 0.672** |
| Education | 0.980 |
| Pro-Socialness | 1.119 |
| Trust in Government | 0.891 |
| Rule Appropriateness | 2.359*** |
| Observed respect for rules | 20.069*** |
| Fear: risk severity | 3.359*** |
| Fear:  risk proximity | 1.352**^.^** |
| Pleasure & Happiness | 1.116 |
| Worsened income position | 1.142 |
|  |  |
| Nagelkerke | 0.162 |
| logLik | -3943.783 |
| McFadden | 0.093 |

*Weighted results ordered logit model with bootstrapping analysis without replacement*
*Sample sizes = 10,000, number of resampling = 10,000. Shown results are average odds ratios over all resamples.* 
*Level of significance: ***p < 0.001, **p <0.010, *p<0.050, p<0.100.*

### Ordered logit analyses

|  | Wave 1 | Wave 2 | Wave 3 |
| --- | --- | --- | --- |
| Age | 1.023*** | 1.023*** | 1.033*** |
| Female | 1.374*** | 1.462*** | 1.859*** |
| Alone | 0.787* | 0.787* | 1.005 |
| Education | 0.992 | 0.972 | 0.849** |
| Pro-Socialness | 1.253 | 1.115 | 1.778* |
| Trust in Government | 0.930 | 0.788**^.^** | 0.477*** |
| Rule Appropriateness | 2.511*** | 2.421*** | 4.114*** |
| Observed respect for rules | 57.020*** | 36.354*** | 12.968*** |
| Fear: risk severity | 3.629*** | 4.159*** | 3.879*** |
| Fear:  risk proximity | 1.283 | 1.389* | 2.310*** |
| Pleasure & Happiness | 1.142* | 1.130* | 1.121 |
| Worsened income position | 1.155**^.^** | 1.184**^.^** | 1.338 |
|  |  |  |  |
| Nagelkerke | 0.184 | 0.176 | 0.244 |
| logLik | -7931.381 | -7647.597 | -4883.575 |
| McFadden | 0.071 | 0.068 | 0.077 |

*Weighted results ordered logit model with bootstrapping analysis without replacement*
*Sample sizes = 10,000, number of resampling = 10,000. Shown results are average odds ratios over all resamples.* 
*Level of significance: ***p < 0.001, **p <0.010, *p<0.050, p<0.100.*
